# Contributions of Beneficial Microorganisms in Soil Remediation and Quality Improvement of Medicinal Plants

**DOI:** 10.3390/plants11233200

**Published:** 2022-11-23

**Authors:** Gang Wang, Ying Ren, Xuanjiao Bai, Yuying Su, Jianping Han

**Affiliations:** Institute of Medicinal Plant Development, Chinese Academy of Medical Sciences & Peking Union Medical College, Beijing 100193, China

**Keywords:** soil degradation, medicinal plants, contamination, microbial bioremediation, growth promotion, quality improvement

## Abstract

Medicinal plants (MPs) are important resources widely used in the treatment and prevention of diseases and have attracted much attention owing to their significant antiviral, anti-inflammatory, antioxidant and other activities. However, soil degradation, caused by continuous cropping, excessive chemical fertilizers and pesticide residues and heavy metal contamination, seriously restricts the growth and quality formation of MPs. Microorganisms, as the major biota in soil, play a critical role in the restoration of the land ecosystem. Rhizosphere microecology directly or indirectly affects the growth and development, metabolic regulation and active ingredient accumulation of MPs. Microbial resources, with the advantages of economic efficiency, harmless to environment and non-toxic to organisms, have been recommended as a promising alternative to conventional fertilizers and pesticides. The introduction of beneficial microbes promotes the adaptability of MPs to adversity stress by enhancing soil fertility, inhibiting pathogens and inducing systemic resistance. On the other hand, it can improve the medicinal quality by removing soil pollutants, reducing the absorption and accumulation of harmful substances and regulating the synthesis of secondary metabolites. The ecological and economic benefits of the soil microbiome in agricultural practices are increasingly recognized, but the current understanding of the interaction between soil conditions, root exudates and microbial communities and the mechanism of rhizosphere microecology affecting the secondary metabolism of MPs is still quite limited. More research is needed to investigate the effects of the microbiome on the growth and quality of different medicinal species. Therefore, the present review summarizes the main soil issues in medicinal plant cultivation, the functions of microbes in soil remediation and plant growth promotion and the potential mechanism to further guide the use of microbial resources to promote the ecological cultivation and sustainable development of MPs.

## 1. Introduction

Traditional Chinese medicine (TCM) is an important part of the medical and health system with a long history [1]. Adequate and high-quality medicinal resources are the foundation of TCM industry development, among which herbal medicine derived from plants plays a pivotal role [2]. The growth and quality formation of medicinal plants (MPs) are closely related to environment, climate, soil, harvest time, biological community and field management measures [3,4,5]. In particular, soil conditions are considered a key factor affecting the agricultural production of MPs [6,7]. However, current soil problems due to continuous cropping, environmental pollution and excessive pesticide residues have seriously restricted the growth and sustainable development of MPs [8,9]. Continuous cropping and monoculture are the main modes of the agricultural industry worldwide. Especially for perennial MPs, such as *Panax ginseng*, *P. notoginseng* and *P. quinquefolius*, it often takes several years from sowing to harvesting. Consecutive monoculture results in soil nutrient imbalance, allelopathic autotoxicity, microbial community change and soil-borne disease increase [10,11]. Furthermore, emissions of industrial waste, indiscriminate use of fertilizers and pesticides and sewage irrigation accelerate soil degradation, resulting in soil hardening, salinization and heavy metal and organic contaminant accumulation [12,13]. According to the national survey bulletin on soil pollution in China, 16.1% of the investigated soil sites were reported to contain excessive levels of pollutants, mainly including eight inorganic pollutants (cadmium, mercury, arsenic, copper, lead, chromium, zinc and nickel) and three organic pollutants (hexachlorocyclohexane, dichloro-diphenyl-trichloroethane and polycyclic aromatic hydrocarbons) [14]. Hazardous chemicals in soil are absorbed and accumulated by MPs and eventually enter human bodies, which threatens clinical safety and poses potential health risks [15,16]. Although some chemical and physical methods have been tried for soil amelioration, they are not very efficient, and the process is complex and expensive [17]. Therefore, other more effective, economical and environment-friendly methods and technologies are needed for degraded-soil remediation to promote the sustainable development of the ecological environment and agricultural production of MPs.

Microorganisms, as major decomposers, are widely distributed in soil, the composition and structure of which are complex and diverse. The dynamic changes of soil microecology mediated by the interactions between plant–microbe–soil communities are ongoing all the time, involving the regulation of soil ecosystems and plant development [18,19]. The core functions of the microbiome in the plant–microbe–soil system are as follows: (1) regulating soil properties and fertility; (2) forming mycorrhizal structures with plant roots; (3) participating in the degradation, fixation and transformation of soil pollutants; (4) inducing systemic resistance of plants; (5) decomposing plant and animal residues in soil; (6) inhibiting the pathogens [20,21,22]. Some beneficial microorganisms, such as *Bacillus*, *Pseudomonas* and *Azotobacter*, have been proven to be of great potential in plant growth promotion and soil remediation [23]. Microbes improve soil fertility and enhance nutrient absorption and utilization of MPs by decomposing plant residues, increasing organic matter content and promoting nutrient availability. Some antagonistic individuals can also degrade and remove pesticides, organic contaminants and heavy metals from the soil, to reduce the accumulation of harmful substances and mitigate the negative impact of abiotic stress on MPs [24,25]. For example, it was reported that after spraying *Paenibacillus polymyxa* five times, the degradation rates of five pesticides (fluazinam, hexachlorocyclohexane, pentachloronitrobenzene, chlorpyrifos and dichlorodiphenyltrichloroethane) in ginseng roots were 66.07%, 46.24%, 21.05%, 72.40% and 54.21%, respectively [26]. Rhizosphere microorganisms, which are in close contact with plant roots, can directly participate in the regulation of plant growth and secondary metabolism via releasing hormones, inhibiting pathogens and facilitating nutrient uptake [27,28].

**Figure 1:**
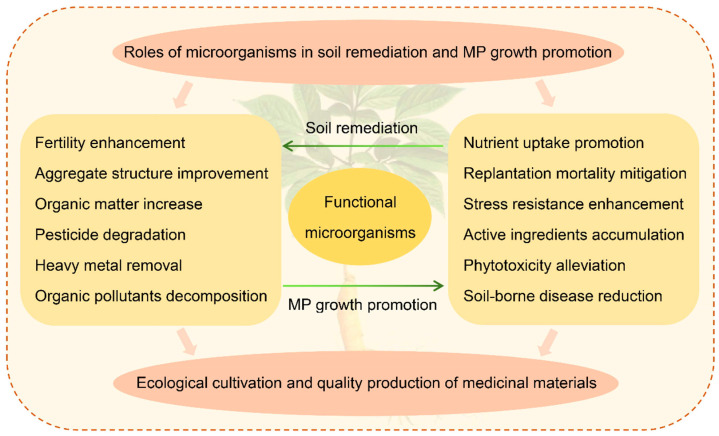
Functions of microorganisms in soil remediation and MP growth promotion.

The microbial biocontrol of degraded soil shows bright prospects in the ecological cultivation and sustainable development of MPs [29,30] (Figure 1). However, medicinal plants are diverse and widely distributed worldwide, with different requirements for the growing environment. At present, the knowledge of how rhizosphere microecology regulates the growth, development and secondary metabolism of different medicinal species is still lacking. More research should be conducted to elucidate the mechanism and signaling pathways of the interaction between soil conditions, root exudates and microbial communities. In this review, we make a comprehensive summary of the main soil issues in medicinal plant cultivation, role of microorganisms in soil remediation and MP growth promotion, and the application prospect of microbial inoculants, to provide reference and guidance for the further utilization of microbial resources in the ecological restoration of degraded soil and the high-quality production of medicinal materials.

## 2. Review Methodology

The review and research articles were retrieved using relevant keywords (involving soil degradation, microecology, contamination, heavy metals, pesticide residues, accumulation, phytotoxicity, oxidative stress, phytoremediation, adsorption, medicinal plants, microbial bioremediation, biodegradation, rhizosphere, nitrogen-fixing bacteria, mineral nutrients, enzymes, growth promotion, quality improvement and active ingredients) from the databases ScienceDirect, Google Scholar, Web of Science, ResearchGate and PubMed. Finally, about 300 articles published mainly from 2010 to 2022 were selected. Then, the useful information related to our topic, such as soil contamination, microbial remediation and growth and quality improvement of MPs, was extracted and summarized from the collected articles according to the criteria followed by Haider et al. [31].

## 3. Soil Problems in Medicinal Plant Cultivation

### 3.1. Continuous Cropping Obstacle

Continuous cropping obstacle refers to the phenomenon that when crops are consecutively planted in the same fields, even under normal management conditions, they will still result in slower plant growth, increased diseases and reduced yield and quality [32,33,34]. The main reasons for this situation include the deficiency and imbalance of soil nutrients, allelopathic autotoxicity and soil microecological changes [10,35]. Soil nutrients are continuously absorbed by plants, followed by fertility reduction. Moreover, the selective uptake of different plants causes an imbalance of nutrient elements in the soil, which gradually fail to meet the demand of their normal growth and development [36]. After two years of *Aconitum carmichaeli* cropping, it was found that the content of total PO_4_^2−^, Ca^2+^, Zn^2+^, Mn^2+^ and Fe^2+^ in soil declined [37]. With the increase in planting time, the yield and active component content (dihydrotanshinone I, cryptotanshinone, tanshinone I and tanshinone IIA) of *Salvia miltiorrhiza* roots were reduced significantly [38]. The yield of *Fagopyrum tataricum* decreased by 6.36%, 24.85%, 78.62% and 83.10% after 1, 2, 3 and 4 years of continuous cropping, respectively, and the available nutrients, soil enzyme activities, number of actinomycetes and content of total chlorophyll and soluble protein in the leaves continuously decreased [39].

The allelochemicals from root exudates enter the soil and surrounding environment, including phenolic acids, organic acids, terpenoids, alkaloids and flavonoids, and some of them have been found to show strong autotoxic effects that inhibit plant growth and development. Guo et al. [40] isolated ten compounds from the rhizosphere soil of *Astragalus hoantchy*, six of which possessed autotoxic activity. Zhang et al. [41] found that the allelochemical, benzoic acid, inhibited root elongation of Arabidopsis seedlings by increasing auxin accumulation via stimulating the expression of auxin biosynthetic genes and *AUX1/PIN2* through the stimulation of ethylene production and an auxin/ethylene-independent ROS (reactive oxygen species) burst. Furthermore, root exudates can change the pH, nutrient availability, C/N ratio and enzyme activity in rhizosphere soil and further affect the balance of rhizosphere microecology [42,43]. Li et al. [44] indicated that under continuous cropping of strawberry, the soil pH significantly decreased, and four phenolic acids, including cinnamic acid, p-hydroxybenzoic acid, ferulic acid and p-coumaric acid, accumulated with time.

Numerous studies have shown that long-term monoculture induced the reduction of beneficial microbes and the increase of pathogens in soil, causing serious soil-borne diseases such as root rot, nematode diseases, damping-off and charcoal rot [45,46,47]. Wei et al. [48] reported that the increase in the relative abundance of pathogenic fungi *Cylindrocarpon*, *Alternaria* and *Fusarium* may be associated with ginseng rusty roots. Soil pH and organic matter content in the rhizosphere of the perennial herb *Atractylodes macrocephala* decreased with cropping time, and *Fusarium* was significantly enriched in the individuals with root-rot disease [49]. Gao et al. [50] also found that in the rhizosphere soil under continuous cultivation of sweet potato, the beneficial fungi *Chaetomium* decreased, while the harmful *Verticillium*, *Fusarium* and *Colletotrichum* increased. In summary, a series of soil problems caused by continuous cropping would severely restrict and hinder the agricultural production of MPs.

### 3.2. Soil Hardening and Salinization

Soil compaction and salinization have become a worldwide issue, especially in arid and semi-arid regions [51,52]. With intensive agriculture increasing, high mechanical load, excessive fertilization and sewage irrigation aggravate soil degradation [53,54]. Globally, there are over 900 million hectares of saline and sodic soils [55], and about 1–2% of soils are being degraded every year due to excessive salinity [56]. The investigation found that in the Yellow River Delta of China, the proportions of soil salinization in 2015 and 2019 were about 76% and 70%, respectively [57]. After excessive application of chemical fertilizers, the contents of K^+^, Na^+^, Ca^2+^, Mg^2+^, NO_3_^−^, HCO_3_^−^ and SO_4_^2−^ in soil increased [58,59]. Phosphorus fertilizers increase the PO_4_^3−^ ion in soil that gradually forms insoluble phosphate with Ca^2+^ and Mg^2+^ [60]. When these elements cannot be quickly absorbed by plants and accumulated in soil, the formation of salinization will be accelerated, which in turn reduces the soil nutrient use efficiency of plants. Elhanafi et al. [61] indicated that nitrogen fertilizers promoted the accumulation of proteins in *Sesamum indicum* seeds, but the oil and soluble sugars presented a significantly low level. The contents of total phenolic and flavonoids with antioxidant activity decreased with increasing N supply. Even for halophytes, the germination and seedling growth are also retarded under salt-stress conditions [62]. Excessive salinity leads to high soil osmotic pressure that can cause physiological water shortage of plants, and even death [63,64]. Plants respond to such stress by accumulating various osmolytes (proline, glycine betaine and sugars), secondary metabolites and antioxidants to maintain cell turgor [65,66,67].

Soil structure and physical properties are destroyed due to the salinization and reduction of organic matter content, followed by the use of heavy-duty machinery in field management, which further hardens the soil [68,69]. Compacted soil weakens the permeability of water and air, hindering the transport and absorption of moisture and nutrients from the soil to plants [70]. Pandey et al. [71] found that soil compaction lowered gas diffusion through the reduction of air-filled pores, thus causing ethylene accumulation in root tissues and triggering hormone responses that impeded plant growth. In addition, the deficiency of oxygen in soil is not conducive to the survival of aerobic microorganisms, while the enriched anaerobic bacteria release hydrogen sulfide with toxic effects on plants [72]. Therefore, establishing effective and economical methods to restore and improve salinized and compacted soils should be of great concern in order to maintain sustainable agricultural development.

### 3.3. Soil Acidification

Soil acidification refers to the process where the base ions are leached and acidic cations (H^+^ and Al^3+^) increase, ultimately resulting in a decrease in soil pH [73,74,75]. The degree of soil acidification is affected by both natural and human factors. SO_2_ and nitrogen oxides in the air and environment settle into the soil with rainfall, accompanied by the loss of mobile sulfate and nitrate anions and soluble alkali cations with rain and irrigation water [76]. In natural conditions without other interference, the self-regulating and buffering capacity of soil will greatly slow down the acidification process. However, the acid deposition is exacerbated by acidic gas emissions such as SO_2_, CO_2_ and NO_2_ from industrial production and human activities, which accelerates the increase of H^+^ and the decrease of soil pH [77,78]. Excessive fertilizer application, especially nitrogen, is thought to be another cause of soil acidification [79]. The nitration and hydrolysis of ammonium sulfate, ammonium nitrate and urea could release lots of H^+^ into the soil. More importantly, excessive fertilization reduces the content of organic matter in soil and weakens its buffer capacity to immediately respond to rapid pH changes [80,81,82]. Long-term continuous cropping also contributes to soil acidification [83,84]. The selective acceptance of nutrients by plants leads to an ionic imbalance in the soil, coupled with the organic acids secreted from roots, which drives pH reduction [85,86]. It was reported that the harvest of aboveground tobacco biomass removed about 339 kg base cations from the soil per hectare per year, which was 7.6 times higher than the anion removal, leading to a 12.5 kmol H^+^ production as the main reason inducing soil acidification [87].

Under acidic conditions, the adsorption ability of soil on Ca^2+^, Mg^2+^, K^+^ and NH_4_^+^ decreases, and they are more easily lost with water, resulting in a decline in soil fertility [88]. Soil acidification impairs phosphorus availability by driving the dissolution of aluminum and iron ions, which will combine with PO_4_^3−^ to form insoluble precipitation [89,90]. Babourina et al. [91] indicated that low pH induced strong H^+^ influx, depolarized plasma membrane potential and led to a significant net K^+^ efflux from the root cell of plants. The bioavailability of Mn, Cd, Cu, Zn, Pb and Cr in soil was enhanced under acidic conditions. Excessive uptake and accumulation of heavy metals can cause toxic reactions in plants, and they eventually enter human bodies through the food chain, posing potential health risks [92,93,94]. Moreover, the structure and diversity of soil microbial communities are also affected by pH changes [95,96]. The increase in soil acidity promotes the enrichment of acidophilic taxa such as Halanaerobiales and Rhodospirillales [97]. Muneer et al. [98] found the relative abundance of *Proteobacteria*, *Actinobacteria*, *Crenarchaeota* and *Firmicutes* was negatively correlated with soil pH, while *Acidobacteria*, *Chlorofexi*, *Bacteriodetes*, *Planctomycetes* and *Gemmatimonadetes* were positively correlated with soil pH. Li et al. [99] revealed that an acidic environment enhanced the toxicity of perfluorooctane sulfonate (PFOS) and chromium (Cr(VI)) to soil bacteria. Acid stress reduced the metabolism of bacteria, while PFOS and Cr(VI) pollution further strengthened the metabolic inhibition involving oxidative stress and cell permeability. In conclusion, acidification has shown a variety of negative effects on soil properties, plant growth and microecological balance.

### 3.4. Contamination of Pesticides, Heavy Metals and Organic Pollutants in Soil

In order to prevent and control plant diseases and insect pests, pesticides (insecticides, fungicides, molluscicides, rodenticides and nematocides) have been widely used around the world for a long time. According to the statistics of the Environmental Protection Agency of the United States, the consumption of organophosphate insecticides alone reached up to 334 million pounds from 2001 to 2007, mainly including chlorpyrifos, dicrotophos, malathion, naled, diazinon, acephate and phosmet [100]. It was found that 90% of fungicides, 60% of herbicides and 30% of insecticides were potentially carcinogenic [101]. As for the pesticide residues in soil, they cannot be quickly decomposed in time, and are absorbed and gradually accumulated by plants. The pesticide-contaminated crops finally enter consumers through the food chain, posing a potential health threat. In addition, the accumulated pesticides in soil migrate with rain and irrigation water, polluting and destroying water sources and ecological environment [102,103].

On the other hand, the release of industrial waste, sewage and domestic garbage aggravates soil deterioration. Heavy metals, pesticides and organic pollutants in these wastes remain and accumulate in soil as high-risk hazardous substances to organisms and ecosystems [104,105,106,107]. The metallic elements can disrupt normal functions of the body’s nerves, kidneys, liver and cardiovascular systems [108]. It has been identified that Cd accumulation in bodies could lead to bone pain and brittle bones, while Pb pollution seriously endangers fertility [109]. Many medicinal herbs have been found containing excessive pesticide residues and heavy metals [110,111,112]. Harris et al. [113] examined the residues of arsenic, cadmium, chromium, lead and mercury in 334 samples of raw Chinese herbal medicines and found that at least one metal was present in the 334 samples and 115 samples accumulated detectable levels of all the tested metals. Maitlo et al. [114] also reported that the contents of heavy metals (Zn, Pb, Cr and Co) in most of the investigated 40 commonly consumed herbal medicines were higher than the maximum allowable limits of WHO.

Heavy metals enter plants to induce oxidative stress and the production of a large number of reactive oxygen species (ROS), which destroy the membrane lipids, proteins, nucleic acids, enzyme activities and various organelles, ultimately resulting in cell death [115,116,117]. Liu et al. [118] revealed that in low temperature conditions, Cd aggravated the destruction of chloroplast ultrastructure and disturbed the ion homeostasis, which also increased ROS accumulation and reduced antioxidant enzyme activities. The CBF-COR signaling pathway was negatively affected by Cd treatment, which reduced the low temperature tolerance of barley. Previous studies have also shown that the soil may be contaminated with heavy metals due to the introduction of fertilizers [119]. For example, phosphorus fertilizer was reported to enrich cadmium in soil, which would interfere with the physiological metabolic activities of plants, such as photosynthesis, gas exchange and nutrient absorption [115,120,121]. Polycyclic aromatic hydrocarbons (PAHs), polychlorinated biphenyls (PCBs) and total petroleum hydrocarbons (TPHs) are the typical organic pollutants in effluent and industrial wastes with high teratogenic, carcinogenic and mutagenic toxicity to humans [122,123]. They are difficult to remove and degrade and thus chronically persist in soil while significantly changing the plasticity, porosity, permeability and water-holding capacity [124,125].

**Figure 2:**
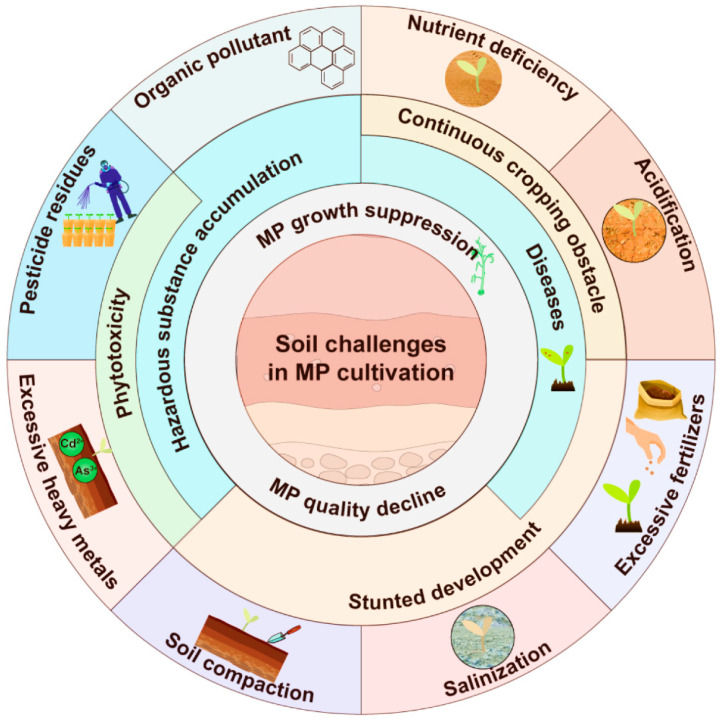
Main soil challenges and their negative effects in the cultivation of medicinal plants. Nutrient deficiency and imbalance, soil compaction, salinization and acidification retard the growth and development of MPs, accompanied by continuous cropping obstacles. The harmful substances, including chemical pesticides, heavy metals and organic pollutants in soil, can cause phytotoxicity, and they are absorbed and accumulated by MPs, posing serious health risks to consumers through the food chain.

The exogenous pollutants mentioned above have shown multiple adverse effects on soil characteristics, plant growth, soil microecology and food safety [126,127,128]. Shen et al. [129] found that heavy metals disturbed the microbial communities in different ways. As and Pb altered the community composition and decreased microbial diversity; Cu reduced bacterial abundance in soil; and Cd and Cr lowered the metabolic capabilities of bacteria. The fungicide Chlorothalonil inhibited the activities of fluorescein diacetate hydrolysis and urease in soil, while Pyraclostrobin inhibited dehydrogenase activity during the exposure period, and both notably changed the diversity and structure of microbial communities [130,131]. Ren et al. [132] showed that in pyrene (a high-molecular-weight PAH)-polluted red soil, the bacteria Chloroflexi, AD3, WPS-2, GAL5, Alphaproteobacteria, Actinobacteria, Deltaproteobacteria and Crenarchaeota were decreased, while Acidobacteria, Betaproteobacteria and Gammaproteobacteria were significantly increased. To sum up, soil contamination has seriously hindered the healthy and sustainable development of agricultural production (Figure 2). It is necessary to take some effective measures to deal with this problem.

## 4. Roles of Microorganisms in Soil Bioremediation and the Potential Mechanisms

**Figure 3:**
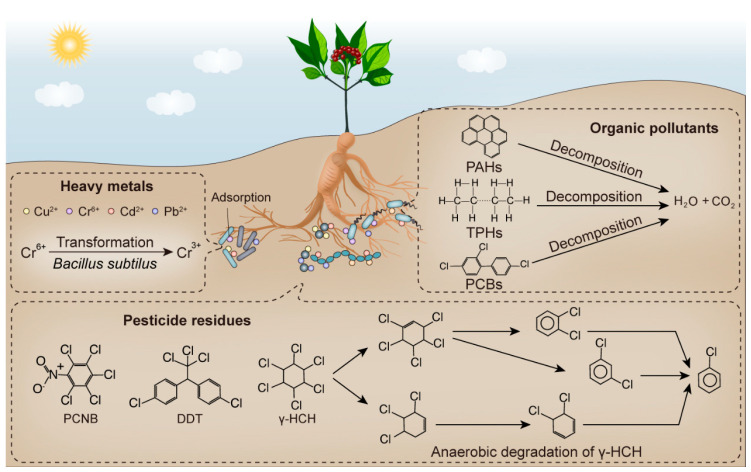
Removal of soil contaminants (pesticide residues, heavy metals and organic pollutants) by microorganisms and the potential mechanism. Heavy metals are adsorbed and fixed on the cell surface by microorganisms. Highly toxic metallic elements can be converted to less or non-toxic substances by redox reactions. The chemical pesticides and organic pollutants are broken down by soil microbes into small molecule compounds, H_2_O and CO_2_.

Soil biota acts as an indispensable factor involved in the restoration of terrestrial ecosystems, and microbiome-dominated biological regulation is an important driving force for the stable development of plant populations. The bioremediation of contaminated soil relies on microbial metabolic activities to remove and degrade the external deleterious chemicals (Figure 3). Furthermore, plant growth-promoting microorganisms also possess a wide range of positive effects to help plants clean up soil pollutants by stimulating plant growth and increasing the bioavailability and absorbability of contaminants to improve the phytoremediation efficacy. Here, we summarize the microbial species with the functions of pollution removal and soil property improvement, including those in MPs and other crops. It is expected that it will guide the use of microbial resources to improve soil conditions before or during the cultivation of MPs (Table 1).

### 4.1. Regulation of Soil Properties and Fertility

Intensive planting of crops may lead to the depletion of soil organic matter and nutrient reserves which, under natural conditions, gradually cannot meet the demand for plant growth and development [179]. Additionally, soil degradation, including salinization, hardening and acidification, has become a great threat to sustainable global agricultural development [180,181]. The microbiota present in the soil is regarded as the game changers in degraded-land restoration [182]. Microorganisms regulate soil properties and fertility through different pathways: (1) microbes can activate soil nutrients and promote their availability; (2) nitrogen-fixing bacteria improve soil fertility by transforming the nitrogen elements; (3) the extracellular secretions of microbes can enhance the stability of soil aggregates; (4) they increase soil organic matter content by decomposing plant and animal residues [133,183,184]. The soil microorganisms with nitrogen fixation function convert N elements in the air and environment into NH_4_^+^-N and NO_3_^−^-N, which are available to plants through ammonification, nitrification, assimilation and denitrification, such as *Rhizobium*, *Azospirillum*, *Burkholderia*, *Nitrosomonas*, *Nitrococcus*, *Nitrobacter*, *Paenibacillus*, *Klebsiella* and *Pseudomonas* [134,185,186]. The nitrogenase encoded by the *nif* gene family is verified as the key enzyme to catalyze the biological N-fixation reaction, which is a two-component system consisting of the separable Fe protein and MoFe protein [187,188,189]. Xie et al. [135] found that *Paenibacillus* was initially incapable of fixing nitrogen, and the N_2_-fixing *Paenibacillus* strains were generated by acquiring the *nif* cluster via horizontal gene transfer from a source related to *Frankia* in early evolutionary history. Rhizobia (*Rhizobium*, *Bradyrhizobium*, *Azorhizobium* and *Mesorhizobium*) have a symbiosis with legume roots to form nodule structures, which under normal conditions can reduce inert N_2_ gas to available ammonia for plant use [190,191,192]. A complex amino acid cycle was found to drive the N-fixation process in legume–*Rhizobium* symbiosis [191].

Some beneficial microbes can also enhance fertility by activating soil nutrients, like *Enterobacter*, *Brevibacillus*, *Mortierella*, *Trichoderma* and *Phyllobacterium* as phosphate-solubilizers, and *Paenibacillus*, *Agrobacterium*, *Acinetobacter* and *Bacillus* for potassium solubilization [23,136,137,193]. In addition, the strains of *Acetobacter pasteurianus*, *Stenotrophomonas rhizophila*, *Curtobacterium* sp. and *Rahnella* sp. have been identified to be capable of both N fixation and P solubilization [183]. The *gcd* gene encoding quinoprotein glucose dehydrogenase is considered the core determinant that governs microbial phosphate solubilization [194,195]. The organic acids and exopolysaccharides released by microbes also help increase the contents of P and K in soil [196,197]. The phosphate-solubilizing microorganisms *Trichoderma asperellum* LZ1 and *Serratia* sp. LX2 were found to improve P availability by reducing soil pH [198]. The tartaric acid, citric acid, lactic acid, malic acid, oxalic acid and gluconic acid produced by potassium-solubilizing bacteria are able to release the fixed K from K-containing minerals [199]. The isolates of *Pantoea agglomerans*, *Rahnella aquatilis* and *Pseudomonas orientali* from paddy rhizosphere soil significantly enhanced the yield and K uptake of crops [138]. Moreover, Jiang et al. [139] reported that the strain *Providencia rettgeri* TPM23 could improve the properties of saline soil. Na^+^ and Cl^−^ contents decreased in TPM23-treated soil, while available N, P and K increased. Furthermore, the activities of alkaline phosphomonoesterases, urease and dehydrogenase were observably promoted by TPM23.

On the other hand, microbes help maintain the stability of soil aggregates through secretions and hyphae networks [182,200]. Extracellular polymeric substances (EPSs), such as polysaccharides, polyuronic and amino acids with adhesive properties from different bacterial species, can bind clay particles to form soil aggregates, thus increasing inter-particle cohesion [201,202]. For example, the glomalin-related soil proteins produced by AMF play the role of particle gluing agents to increase soil aggregate stability, which supports resistance to erosion, carbon storage and water-holding capacity [203,204]. Meanwhile, the hyphae of fungi and actinomycete entangle particles to form a network to further stabilize soil structure [201]. By decomposing plant and animal residues, microbes help increase the content of organic matter, which can improve soil fertility retention capacity and buffer performance [205,206]. The necromass of microbes themselves is also an important source of soil organic carbon stock and is governed mainly by fungal necromass carbon [207]. In conclusion, soil microorganisms can not only activate nutrients to directly improve fertility, but also enhance the capacity of water storage and fertilizer conservation by regulating soil characteristics.

### 4.2. Degradation of Pesticides and Organic Pollutants in Soil

Chemical pesticides and other organic pollutants such as PAHs, PCBs and TPHs are introduced into farmlands and accumulate in soil because they are difficult to break down rapidly [123,208]. Fortunately, microorganisms can decompose these hazardous compounds and make outstanding contributions to agricultural production, environmental protection and human health. For example, the *Microbacterium* sp. D-2 isolated from dicofol (an organochlorine insecticide)-contaminated agricultural soil presented an effective dicofol-degrading function, which could degrade 85.1% of 50 mg/L dicofol within 24 h [143]. Soil microbes remove the contaminants mainly through biodegradation and enzymatic mineralization [209]. They can convert the refractory organic macromolecules into water, carbon dioxide and less toxic compounds [210]. The strains of *Pseudomonas*, *Trichoderma*, *Sphingomonas*, *Paenibacillus*, *Bacillus*, *Acinetobacter*, *Stenotrophomonas*, *Agrobacterium*, *Alcaligenes*, *Burkholderia*, *Serratia*, *Klebsiella*, *Streptomyces*, *Enterobacter*, *Rhizobium* and *Xanthomonas* have been identified with the ability to break down the xenobiotics [132,144,145,211,212]. The soil bacteria *Pseudomonas putida* and *Acinetobacter rhizosphaerae* were found to be able to hydrolyze both organophosphate and carbamate pesticides [146]. A novel glyphosate-degrading species, *Chryseobacterium* sp. Y16C, was isolated from soil, which could completely degrade the herbicide glyphosate at 400 mg/L concentration within four days [147]. He et al. [168] reported that *Hyphomicrobium* sp. GHH, in combination with the cultivation of *Lolium perenne,* posed a great potential for remediating the soil contaminated by 17α-ethynylestradiol, a typical environmental endocrine-disrupting chemical. The *Arthrobacter* sp. strain HB-5 demonstrated excellent atrazine removal capacity, and the degradation half-life in HB-5 inoculated soil was three times less than that in natural soil [148]. Besides, the strains of *Klebsiella pneumonia* PL1, *Pseudomonas mendocina*, *Brevundimonas olei*, *Serratia marcescens*, *Sphingobium yanoikuyae* B1 and *Pseudomonas* sp. USTB-RU have also been shown to decompose organic pollutants such as pyrene, benzo(a)pyrene, phenanthrene and naphthalene [169,170,171,172]. The combination of biosorption and biodegradation of *Pseudomonas* sp. SDR4 and *Mortierella alpina* JDR7 help achieve a remarkable reduction of PAHs [173].

The degradation of soil pollutants by microorganisms is regulated by gene expression and multiple enzymes. Cytochrome P450 genes (*CYPs*) are involved in encoding a large superfamily of heme-thiolate proteins, which catalyze the exogenous and endogenous compounds through chemical modifications and stereotactic oxidation [213]. Cyp enzymes participate in the degradation of heterologous substances via primary and secondary metabolic processes [214]. Chadha et al. [215] identified 477 cytochrome P450s from *Trichoderma* spp. with the potential for environmental pollutant degradation. The Ta*Cyp*548-2 of *CYPs* from *Trichoderma atroviride* T23 was found to reduce the chlorinated organophosphate-based pesticide, dichlorvos, by two steps, i.e., production of the intermediate product, 2,2-dichloroethanol, and then conversion of 2,2-dichloroethanol to less toxic 2,2-dichloroethanol acetate [149]. The dehalogenase, dehydrogenase, dehydrochlorinase, esterase, phosphatase, salicylate hydroxylase, paraoxonase, and dioxygenase from soil microorganisms accelerate the removal of pesticides and high molecular weight organics [101,210,216]. Bacterial enzymes, like organophosphorus hydrolase, methyl parathion hydrolase and OpdA regulated by *opd*, *mpd* and *opdA* genes, respectively, are involved in the preliminary hydrolysis of organophosphorus pesticides [150,151]. Aswathi et al. [152] indicated that the organophosphate hydrolase of *Pseudomonas nitroreducens* AR-3 eliminated 42% of 100 mg/L chlorpyrifos in just 2 h. The oxygenases, encoded by *nidA*, *nidB*, *nidA3*, *nidAB*, *nahAc*, *nagAc*, etc., are considered key enzymes to drive the initial dihydroxylation step of aromatic rings to promote the decomposition of PAHs [174,175,217]. The salicylate hydroxylase, catechol 1,2-dioxygenase, 2-carboxybenzaldehyde dehydrogenase and catechol 2,3-dioxygenase expressed in *Pseudomonas aeruginosa* PSA5 and *Rhodococcus* sp. NJ2 were found to catalyze the degradation of benzo(a)pyrene [176].

However, some pollutants are not suitable as the sole substrates for microbial growth, such as high molecular weight polyaromatic hydrocarbons, aliphatic and aromatic polychlorinated organics, which are not normally biodegradable. Soil microorganisms transform and use such molecules through cometabolism, namely assimilating other growth substrates together with these non-growth substrates [218]. Microbial cometabolism can achieve the biological transformation of a non-growth substrate with non-specific enzymes, the synthesis of which, in microbial cells, can only be induced by growth substrates that provide energy for cell growth and maintenance [153]. For example, the cometabolic bioregeneration of activated carbons derived from the removal of 2-chlorophenol by using phenol as the growth substrate [219]. Benzene and toluene degradation of *Pseudomonas oleovorans* DT4 were greatly enhanced by tetrahydrofuran acting as an “energy generator” [177]. The synergy between fungi and bacteria promoted the PAH mineralization to CO_2_, and lignin stimulated the co-metabolic biodegradation of benzo(a)anthracene by recruiting the bacterial taxa *Methylophilaceae* and *Sphingomonadaceae* [220]. Collectively, microbial communities have great potential in the remediation of pesticides and organic pollutant-contaminated soil.

### 4.3. Removal of Heavy Metals from Soil

Heavy metals, mainly including As, Cd, Zn, Pb, Mn, Cr, Cu and Hg, cause potential phytotoxicity to MPs, such as oxidative damage, interfering enzyme activities, membrane damage, stomatal closure, photosynthesis reduction and carbon metabolism retardation, which is considered detrimental to seed germination and plant growth [221,222,223]. Bioremediation involving soil microorganisms is proposed as a cost-effective and environmentally friendly method to rehabilitate land contaminated by heavy metals to speed up the recovery of ecosystems and biodiversity [224]. The indigenous fungal strains *Aspergillus fumigatus* and *A. terreus* isolated from contaminated soil showed excellent Pb and Hg removal capability [225]. Meanwhile, *Aeromonas*, *Bacillus* and *Pseudomonas* act as important organisms in the remediation of heavy metal-contaminated soil [226]. Microbes can collect heavy metals in soil by biosorption. Some ions in functional groups on the cell surface, such as oxygen, nitrogen, sulfur and phosphorus, can be complexed with metal ions as coordination atoms [109]. The phytochelatins, glutathione, metallothionein and glomalin generated by AMF can also immobilize metals and promote the adaptation of host plants to stressful environments. Altowayti et al. [160] illustrated that the arsenic-resistant *Bacillus thuringiensis* strain WS3 could effectively achieve 10.94 mg/g of As (III) removal via adsorption processes at optimum conditions. This process is often accompanied by ion exchange. It was demonstrated that *Saccharomyces cerevisiae* released approximately 70% of K^+^ and 60% of Mg^2+^ during Cu^2+^ adsorption [227].

Furthermore, soil microorganisms enhance the phytoremediation of contaminated soil by alleviating the phytotoxicity of heavy metals and promoting their uptake by plants. Rhizosphere exudates, such as organic acids, accelerate the solubilization of metals to increase bioavailability [161,228]. Hussain et al. [162] showed that the synergy of *Kocuria rhizophila* and citric acid increased plant biomass by 38.73% and the accumulation of Cd, Cr, Cu and Ni by 40.63%, 56.39%, 59.1% and 39.76%, respectively. The manganese-tolerant strains *Bacillus cereus* HM5 and *B. thuringiensis* HM7 promote Mn absorption of *Broussonetia papyrifera*, whose concentration increased in the aerial parts of plants. Additionally, the two *Bacillus* spp. mitigated Mn-induced oxidative stress by reducing malonaldehyde content and antioxidant enzyme activities in leaves [163]. Kumar et al. [164] found that the isolate *Pseudomonas lurida* EOO26 presented multi-metal tolerance, drought resistance and plant growth-promoting attributes, and the inoculation with EOO26 increased Cu uptake by 8.6 times in roots and 1.9 times in leaves of *Helianthus annuus* than uninoculated individuals. Microorganisms can also drive redox reactions to change the valence of heavy metals and reduce their biological toxicity [109]. For example, *Bacillus subtilus* MAI3 could reduce highly toxic Cr (VI) into less soluble Cr (III) in soil by producing chromium reductases and antioxidants, which improved the growth and photosynthesis of plants [165]. In summary, microorganisms can serve as powerful tools for the remediation of heavy metal-contaminated soil through multiple pathways.

## 5. Function of Soil Microorganisms in Growth Promotion and Quality Improvement of Medicinal Plants and the Potential Mechanisms

Medicinal plants are subjected to a variety of biotic and abiotic stresses throughout their growing period. Soil microbes are known as the second genome of plants, whose structure and functions to host plants dynamically change with stress and environmental stimuli. The growth-promoting microorganisms improve the growth and quality of MPs by accelerating nutrient absorption, enhancing stress resistance, inhibiting pathogenic organisms and regulating secondary metabolism. We have summarized the positive role of beneficial soil strains/microbes in the production of different medicinal species in Table 2. An in-depth understanding of plant–microbe interactions will undoubtedly contribute to the production of high-yield and high-quality medicinal resources and lay a foundation for the vigorous development of traditional Chinese medicine industry.

### 5.1. Microorganisms Enhance the Environmental Stress Resistance and Growth of Medicinal Plants

Soil microbial diversity plays a crucial role in securing stable plant production in global ecosystems and buffering against extreme climate events [272]. The microflora can promote the growth and development of MPs mainly through the following channels: (1) improving soil physical and chemical properties to provide a suitable growth environment for MPs; (2) activating soil nutrients and increasing their availability; (3) symbiosis with plant roots to form mycorrhizal structure in order to increase the contact with soil and promote the uptake of water and nutrients; (4) inducing systemic resistance and enhancing the adaptability of MPs to environmental stress [139,273,274]. Root-associated microbes that participate in optimizing N, P and K capture are critical for plant growth and nutrient acquisition [275,276]. The native phosphate solubilizing strains *Bacillus subtilis* PSB-1 and PSB-36 could significantly improve the P-availability in semi-arid saline soil and increase the yield and essential oil content of *Foeniculum vulgare* seeds [253]. The combination of P fertilizers and biofertilizers (AMF and *Pseudomonas florescent* bacterium) was found to promote water absorption of *Echinacea purpurea* under drought stress and transfer more P elements from roots to leaves [250]. The treatment with *Bacillus amyloliquefaciens* EZ99 and sucrose amendments facilitated the potassium utilization in rhizosphere soil and increased the root fresh weight of the medicinal herb *Rheum palmatum* [254]. The sesquiterpene compound, cedrene, from *Trichoderma guizhouense* NJAU4742, was reported to efficiently promote plant growth and suppress soil-borne pathogens. It could also require the TIR1 and AFB2 auxin receptors, IAA14 downstream auxin-responsive protein and ARF7 and ARF19 transcription factors to stimulate lateral root development [277]. AMFs are recognized as beneficial symbionts of most land plants that can strengthen plant nutrient uptake. It was indicated that mycorrhizal colonization strongly induced the expression of nitrate transporter genes *OsNPF4.5*, *ZmNPF4.5* and *SbNPF4.5* in roots, which served as the drivers of mycorrhizal NO_3_^−^-N acquisition [278]. In addition, Chen et al. [279] revealed that the DNA methylation modifications induced by plant growth-promoting bacteria mediated the promotion process in roots, and the epigenetic modifications remained functional after the elimination of the inoculum from the microbiome.

Microbial communities can also maintain the normal growth of MPs by alleviating the adverse effects caused by continuous cropping obstacles. The inoculation with *Bacillus subtilis* 50-1 isolated from soil made *Panax ginseng* replanting mortality and pathogenic *Fusarium* abundance decrease by 63.3% and 46.1%, respectively [229]. Phenolic acids are one of the main allelopathic autotoxic substances that result in replanting problems of many MPs [280]. It has been reported that phenolic acids are positively associated with beneficial *Pseudomonas*, *Streptomyces*, *Nitrobacter*, *Nitrospira* and *Bacillus* in rhizosphere soil [281]. Gauri et al. [282] suggested that the *Azotobacter* sp. strain SSB81 could degrade the accumulated phenolic acids by oxidative and non-oxidative pathways to reduce the toxic level and increase soil fertility. Meanwhile, *Pseudomonas aeruginosa* with catalpol-degrading capacity was considered to have great potential in mitigating the autotoxicity of medicinal *Rehmannia glutinosa* [283].

Ethylene is an important phytohormone known to regulate fruit ripening, leaf abscission and plant senescence. However, ethylene at high concentrations triggers the inhibition of root and stem growth together with premature senescence, leading to poor plant performance [284]. Stress conditions can induce high levels of ethylene in plants and halt root elongation and nitrogen fixation [21]. Sadeghi et al. [285] found that water deficiency led to ethylene accumulation in leaves, lowering the biomass, leaf area and plant height of the medicinal herb *Cichorium intybus*. 1-aminocyclopropane-1-carboxylate (ACC) is the vital precursor for ethylene synthesis. Numerous studies have confirmed that plant growth-promoting microbes produce ACC deaminase to equilibrate the ethylene content to an optimum level in plants. ACC deaminase catalyzes the cleavage of ACC to ammonia and α-ketobutyrate to facilitate plant growth and development under environmental stresses, such as flooding, drought, high temperature, cold, radiation and insect predation [286,287,288]. *Bacillus subtilis* LDR2 alleviated the ethylene-induced damage under drought conditions and enhanced nodulation and AMF colonization to improve nutrient uptake and growth of *Trigonella foenum-graecum* [251]. The rhizobacteria *Staphylococcus sciuri*, *Zobellella denitrificans* and *Arthrobacter endophyticus* improved photosynthesis of *Pistacia vera* subjected to salinity and drought stresses and significantly increased the shoot and root dry weight, leaf number, leaf area, shoot and root K^+^ concentration, and relative water content [248]. It was also illustrated that the halophilic and halotolerant bacteria from salt-contaminated soil belonging to *Bacillus*, *Staphylococcus*, *Oceanobacillus*, *Exiguobacterium* and *Halobacillus* could enhance plant growth under salt stress [289].

### 5.2. Microorganisms Promote the Accumulation of Active Ingredients in Medicinal Plants

The content of active ingredients is considered the key to determining MPs’ clinical efficacy. Among them, secondary metabolites are the main substances with pharmacodynamic functions [290,291]. Chen et al. [246] demonstrated that rhizosphere microbiota was closely related to the contents of oxymatrine, sophoridine and matrine in *Sophora flavescens* roots. Microbes can synthesize and secrete chemical molecules, which are called elicitors, to trigger the plant defense responses to biotic or abiotic stress [292,293]. For instance, Pankaj et al. [249] suggested that the efficient halotolerant isolates *Pseudomonas plecoglossicida* (KM233646), *Acinetobacter calcoaceticus* (KM233647), *Bacillus flexus* (KM233648) and *B. safensis* (KM233652) strikingly improved the growth and bacoside A yield of medicinal *Bacopa monnieri* planted in natural salt-affected soil. Artemisinin production in *Artemisia annua* roots was increased after the elicitor treatment of mycelial extracts from *Colletotrichum* sp. [243]. Microbial elicitors, including proteins, oligosaccharides and polyunsaturated fatty acids, can bind to plant cell receptors to activate the signaling pathways that induce the secondary messenger production and transcription factor activation, which further promote the gene expression of specific enzymes to regulate the secondary metabolites synthesis [294]. The elicitor-mediated secondary metabolites cover a wide range of chemical components, such as flavonoids, terpenoids, alkaloids, tannins and phenolic acids [265,295]. The polysaccharide-protein fractions from the rhizobacterium *Bacillus cereus* significantly stimulated hairy root growth and tanshinone accumulation of *Salvia miltiorrhiza* [240]. *Pseudomonas fluorescence* elicited the production of hyoscyamine and scopolamine in *Hyoscyamus niger* and hypericin and pseudohypericin in *Hypericum perforatum* [266,268]. *Aspergillus niger*, *Coriolus versicolor* and *Ganoderma lucidum* were used as the elicitors to enhance the salidroside synthesis of *Rhodiola sachalinensis* hairy roots [296].

Previous studies have also shown that microorganisms promote the formation of secondary metabolites in MPs by facilitating the uptake of mineral nutrients, especially nitrogen and phosphorus [297]. For example, tyrosine and phenylalanine are important precursors of rosmarinic and caffeic acids. AMF symbiosis can catalyze amino acid synthesis by promoting N absorption to drive the accumulation of specific metabolites [264]. *Funneliforms mosseae*, *Glomus mosseae* and *G. veriforme* were observed to improve P acquisition of *Glycyrrhiza uralensis* and promote proline accumulation and glycyrrhizin concentration in stems and roots [236,237]. The inoculation with *Claroideoglomus etunicatum* or *Gigaspora albida* increased total foliar phenols and tannins in the Brazilian medicinal species *Commiphora leptophloeos* [263]. Additionally, microbes could change and optimize the chemical composition of MPs. *Glomus intraradices* altered the relative quantity of essential oil patterns and significantly increased bornyl acetate, 1,8-cineole, α- and β-thujones in *Salvia officinalis* [242]. Li et al. [256] found that bacterial and fungal community composition around the rhizosphere varied over the cultivation years of *Astragalus mongholicus*. The richness of *Stenotrophomonas* was positively correlated with astragaloside content, while *Phyllobacterium* and *Inquilinus* were positively correlated with calycosin content in roots. In conclusion, microorganisms promote the accumulation of active ingredients by eliciting the secondary metabolism of MPs, thus enhancing pharmacological efficacy.

### 5.3. Soil Microorganisms can Enhance the Disease Resistance of Medicinal Plants

Disease and insect pests are another great challenge that seriously inhibits the growth of MPs, especially in perennials. For example, *Panax ginseng* is prone to root rot, root rust, black spot, grey mold, root-knot nematodes and so on during a generally more than a 4-year growing period, causing low yield and poor quality [48,230]. Microbial species improve plant health condition by enhancing their defense system, which triggers the induced systemic resistance (ISR) by regulating the salicylic acid, abscisic acid, jasmonic acid, ethylene and hormonal signaling pathways [298,299]. The functional genes participating in detoxification, biofilm formation and plant-microbiome signaling pathways are significantly enriched in diseased plants [300]. The expression of jasmonic acid- and ethylene-regulated genes, including *Lipoxygenase 2*, *Plant defensin 1.2* and *Hevein-like protein,* were strengthened by the treatment of rhizobacteria, which was verified to enhance the ISR process [301]. ISR stimulates the host defense response to protect plants from bacterial and fungal pathogens, root-knot nematodes, blue mold, damping off and systemic viruses [302,303]. The beneficial soil fungus *Mortierella verticillata* NRRL 6337 was found to exert highly potent anthelmintic activities that could efficiently shield the host from nematode attacks [304]. The small and cysteine-rich proteins secreted by *Trichoderma virens* enhanced the symbiotic relationship between soil microbes and plants. The molecules served a positive role in supporting plant’s defense against parasites as well as pathogens [305]. Photosynthetic bacterium *Rhodopseudomonas palustris* GJ-22 improved *Nicotiana benthamiana* growth by producing indole acetic acid and 5-aminolevulinic acid, which also strengthened plant resistance against tobacco mosaic virus by priming pathogenesis-related genes [306]. Patel et al. [307] indicated that the lipopolysaccharide from *Alcaligenes faecalis* was a potential biocontrol agent to enhance plant immune response to fusarium wilt.

On the other hand, more and more evidence has suggested that plant rhizosphere recruits beneficial microbes to suppress soil-borne pathogens [308]. Host plants attract beneficial microbes through the modulation of plant-microbiome signaling pathways, which evolve LysM receptors to recognize and parse microbial elicitors and trigger intracellular signaling to restrict or facilitate microbial colonization [309]. Yuan et al. [310] showed that the incorporation of pineapple residues in soil increased antagonistic fungal richness to alleviate the pathogen pressure. The signaling molecules in root exudates serve as a link for plant–microbe communication, which could induce the microbiota to respond to the environment and the host states. Glutamic acid, either secreted by plants or added exogenously, could protect plants against pathogens by reshaping the core microbial community. The supply of glutamic acid increased the abundance of beneficial populations of *Streptomyces*, Bacillaceae and Burkholderiaceae and reduced pathogenic *Botrytis* and *Fusarium* to control and alleviate diseases [311]. The transporters control the root-to-soil delivery of specialized metabolites to manipulate the rhizosphere microbiota and thereby affect plant fitness. For example, cucurbitacins, synthesized by operon-like gene clusters, are the bitter triterpenoids peculiar to cucurbit plants. Two Multidrug and Toxic Compound Extrusion (MATE) proteins were verified to be involved in the transport of cucurbitacins from roots into the soil to modulate the rhizosphere microbiome by selectively enriching *Enterobacter* and *Bacillus*, which in turn triggered robust resistance against the wilt fungal pathogen *Fusarium oxysporum* [312].

The aggregated beneficial microorganisms around plant rhizosphere also help restrict the access of harmful pathogens whose growth and survival are inhibited by specific microbes with antibiotic properties [313]. The plant growth-promoting *Bacillus* spp. were identified to increase the abundance of potentially beneficial bacterial genera *Sphingopyxis*, *Sphingomonas*, *Lysobacter*, *Nitrospira*, *Bradyrhizobium*, *Chitinophaga*, *Pseudomonas*, *Dyadobacter*, *Gemmatimonadetes*, *Streptomyces* and *Rhizomicrobium*, and fungal genera *Cladosporium*, *Cladorrhinum* and *Aspergillus*, accompanied by reducing potentially pathogenic *Fusarium* and *Talaromyces* in the rhizosphere [314,315]. The lytic enzymes (e.g., glucanase and cellulase) from antagonistic bacteria can destroy the cell membranes of pathogenic fungi, and *Bacillus* species secrete lipopeptides (fengycin, surfactin and iturin) to block the growth and colonization of pathogens [316]. The ACC deaminase-containing *Pseudomonas putida* (WPTe) prevented *Papaver somniferum* from downy mildew and significantly promoted growth and yield [259]. AMF biofertilizers alleviated replanting diseases of American ginseng by reducing deleterious *Fusarium oxysporum*, *F. solani* and *Candidatus Solibacter* [235]. Furthermore, Jiang et al. [317] revealed that the rhizosphere edaphon of resistant varieties could recruit distinct bacterial taxa associated with disease suppression. It was advocated that microbial transplantation from resistant donors should be promising to modulate soil microecology and plant health.

### 5.4. Microbes Alleviate the Toxicity and Accumulation of Soil Pollutants in Medicinal Plants

The accumulation of pesticides, heavy metals and other organic pollutants in soil has become a serious ecological problem. Large quantities of these harmful substances pose a severe threat to humans and other organisms in the environment, and their residues in medicinal materials adversely affect clinical safety. Soil microorganisms can act as the restorers of contaminated soil, removing the exogenous chemicals and reducing their accumulation and toxicity in plants [318,319,320]. The enzymatic systems, including hydrolytic enzymes, esterases, nitrilases, oxygenases, dehalogenases amidases, and carbon–carbon lyases, facilitate the biodegradation of pesticides and organic macromolecular pollutants to non-toxic or low toxic small molecules [321]. *Enterobacter ludwigii* sp. CE-1 was reported to rapidly transform the herbicide chlorimuron-ethyl with lasting toxicity into 2-amino-4-chloro-6-methoxypyrimidine and non-toxic saccharin, decreasing the phytotoxicity and inhibition to plant growth [322]. The bacterial strain *Pseudomonas* sp. RPT 52 isolated from agricultural fields could catalyze the metabolism of three different chlorinated pesticides, imidacloprid, endosulfan and coragen, with a toxicity reduction of the parent compounds [323]. Soil microbiome also moderates the suppression of toxic pollutants on physiological metabolism by reducing their residues and accumulation in MPs. Tripathi et al. [261] demonstrated that *Bacillus* sp. CIMAP-A7 significantly reduced atrazine content in the important medicinal plant *Andrographis paniculata* and ameliorated the induced oxidative stress, the inoculation of which increased the content of total chlorophyll, carotenoid, proteins and secondary metabolites. The mixed microbial culture PCS-1 from continuous cropping fields was able to degrade seven kinds of pesticides and reduce their residues in the roots, stems and leaves of *Medicago sativa*. *Pseudomonas*, *Enterobacter*, *Aspergillus* and *Rhodotorula* were determined to be the dominant genera with biodegradation ability in PCS-1 [324].

Metal-antagonistic and tolerant bacteria are recruited and enriched in rhizospheres to alleviate the phytotoxicity caused by heavy metals and maintain the normal growth of plants. The rhizobacteria scavenge ROS and avert the oxidative stress induced by heavy metals via stabilizing malondialdehyde content and enhancing the gene expression and activities of antioxidant enzymes, such as catalase, peroxidase and superoxide dismutase [325]. Glutathione (GSH) is another crucial non-enzymatic antioxidant to remove ROS in plants via sulfhydryl groups, which can also directly chelate metals to form GSH-metal complexes for detoxification. It was suggested that *Bacillus altitudinis* WR10 derived the down-regulation of Glutathione S transferases gene expression for a high GSH level in response to metal stress. Furthermore, WR10 regulated phenylpropanoid biosynthesis that might promote phenolic acid production for protecting plant cells from metal toxicity [326]. Aluminum stress stimulated the enrichment of *Bacillus*, *Pseudomonas*, *Arthrobacter* and *Serratia* that mitigated Al-toxicity and bacterial wilt to *Zingiber officinale* in especially acidic soil [260]. Wei et al. [241] found the microbial inoculant and garbage enzyme greatly reduced Cd absorption of *Salvia miltiorrhiza*, with the accumulation of total tanshinones increasing. Additionally, Chen et al. [327] revealed two potential mechanisms of AMF-mediated arsenate resistance, i.e., AMF colonization may restrict the phosphate/arsenate transport system in roots to reduce As uptake, and AMF may accelerate As efflux from mycorrhizal roots.

In summary, the microbiome plays an important role in degraded-soil restoration and MP growth promotion. However, it has to be admitted that biological regulation based on soil microorganisms also has some limitations. Firstly, it is a time-consuming process. In the early stage, microbial communities need to undergo a long course of colonization, proliferation and physiological metabolic activities to gradually improve soil properties. As for pollutant removal, not all the materials in the soil can be absorbed and transformed by microorganisms. Some harmful substances are not bioavailable or are also toxic to microbes [219]. Accordingly, to further enhance the effectiveness of land improvement, microbial resources may be used in combination with other soil amendments such as organic fertilizers and biochar [166]. In addition, the composition and structure of microbial communities are affected by surrounding environment and root exudates of MPs. Some beneficial bacteria may only exist and survive in certain specific medicinal species. More studies should be conducted to explore the effects of rhizosphere microecology on different MPs so as to better exploit and utilize microbial resources for specific objects.

## 6. Development and Application of Microbial Inoculants for Medicinal Plants

Microbial inoculants, referring to products consisting of proven beneficial microorganisms, have been recommended for artificial addition to the soil in field management [248,328]. The dominant bacteria in the products mainly include *Pseudomonas*, *Bacillus*, *Burkholderia*, *Azotobacter*, *Azospirillum*, *Paenibacillus* and so on [254,282,329]. These microbial individuals with rapid reproduction, strong vitality, safe and non-toxic characteristics can quickly colonize plants and soil, which occupy a dominant position to resist other pathogens [330,331]. The treatment with *Bacillus amyloliquefaciens* HK34 effectively induced systemic resistance in *Panax ginseng* against *Phytophthora cactorum*, the main pathogen causing foliar blight and root rot [231]. Sun et al. [332] proposed that the inoculant *Bacillus velezensis* SQR9 recruited indigenous beneficial bacteria *Pseudomonas stutzeri* in the rhizosphere to promote plant growth, and the synergistic biofilm formation helped plants relieve salt stress. The branched-chain amino acid biosynthesis pathways were suggested to contribute to the syntrophic cooperation between SQR9 and *P. stutzeri*.

The development of microbial agents is generally in accordance with the following steps: (1) analysis of soil microbial composition and structure; (2) screening, isolation and identification of plant growth-promoting strains; (3) preparation of microbial seed fluid; (4) formation of microbial agent products; (5) field experiments to test the inoculation effect [154,232]. It has also been found that microbial consortium sometimes performs better than a single strain owing to the stable and comprehensive metabolic function [324]. For example, different microbial species have their own specific pesticide degradation spectrum covering only one or several pesticides. Individual microorganisms cannot evolve a full-scale metabolic mechanism to cope with multiple chemical compounds. Under such circumstances, the mixed microbial system shows greater advantages in the complete degradation of toxic molecules relying on the synergistic effect among various strains [26,333]. The bacterial consortium of *Raoultella* sp. XY-1 and *Pandoraea* sp. XY-2 isolated from tetracycline-contaminated soil presented better growth improvement and tetracycline degradation efficiency compared with the single individuals [178]. Li et al. [155] also showed that microbial consortia were more efficient than single degrader strains in the clean-up of organic chemicals such as isoproturon in soil. A positive association was revealed between the phylogenetic patterns of biosynthetic gene clusters (BGCs) and phylogenetic distance within *Bacillus*. The targets with closer genetic distance tended to share more BGCs, and the antagonism intensity was positively correlated with the phylogenetic distance and BGC distance between strains Xia et al. [334]. These findings offer a deeper insight into the driving force and intrinsic mechanism of microbial interactions, which is of great significance in guiding the design of synthetic microbial communities for practical purposes. The compatibility and synergy of *Glomus mosseae* and nitrogen-fixing *Bacillus subtilis* were indicated to dramatically enhance the growth, biomass yield and content of secondary metabolite artemisinin of *Artemisia annua* [245]. To sum up, microbial agents can be used as an excellent alternative to chemical fertilizers and pesticides to maintain the ecological cultivation of MPs and the sustainable development of the traditional Chinese medicine industry.

## 7. Conclusions and Future Prospects

Sufficient and high-quality medicinal materials are the basis for promoting health industry development and safeguarding people’s life safety. However, soil issues caused by continuous monoculture, excessive and long-term application of chemical fertilizers and pesticides and exogenous pollutants have become a serious ecological problem and restrict the growth and quality formation of MPs. Microbial bioremediation has attracted more and more attention because of the advantages of economic efficiency, harmless to environment and non-toxic to organisms. As ideal alternatives to conventional fertilizers and pesticides, the introduction of beneficial microbes has a bright application prospect in repairing degraded soil and improving the growth and officinal value of MPs. Nevertheless, current concerns about soil–plant–microbe interactions are mainly focused on food crops such as soybean, rice, maize and wheat. Compared with these crops, the species of MPs are more diverse and complex, but the mechanism research of microbial influence on their growth and pharmacological effects is still lacking. In addition, some beneficial microorganisms were validated as biocontrol agents only under laboratory conditions, but most of them have not been widely popularized and applied in large-scale agricultural production. Therefore, to better exploit and utilize the microbial resources in soil, future studies aimed at the interactions between MPs and soil microbes can be conducted in the following areas: (1) establishing microbial germplasm banks to lay the foundation for the collection, classification, preservation and further application of microbial resources; (2) isolating and identifying the core microbial species with the functions of pollutant removal and pesticide degradation in soil as potential bioremediation agents; (3) exploring the mechanism of microorganisms enhancing the growth, stress resistance and disease resistance of MPs to promote the sustainable development of traditional Chinese medicine industry; (4) elucidating the signaling pathways of soil microbes regulating the secondary metabolism of MPs in order to facilitate the production of medicinal materials with high and stable content of active ingredients; (5) developing more efficient plant growth-promoting microbial inoculants for different medicinal species to produce high-yield and high-quality herbs. Furthermore, the development of metagenomics, metabolomics, proteomics, transcriptomics and other omics technologies provides strong support for the in-depth exploration of the interaction mechanism and signaling pathway in the soil–microbial–medicinal plant system through multi-method combinations. The correlation analysis between plant metabolites and metagenomes of rhizosphere microbes can reveal the effects of toxic allelopathic substances secreted by roots on soil microecology. Exploring the interaction between chemical signals and microbial communities can help clarify the regulatory mechanism of reducing successive cropping obstacles by the microbiome. Furthermore, soil amendments and biological control can be applied to guide the amelioration of the rhizosphere environment of medicinal plants so as to improve their yield and quality. It can be predicted that microbial resources in the soil will act as a powerful driving force in ecological restoration and promote the production of high-quality medicinal materials.

## Figures and Tables

**Table 1:** Functional microorganisms and their contributions to soil remediation.

Soil Issues	Strains/Microbes	Contributions	Reference
Nutrient deficiency and imbalance	*Pseudomonas libanensis* EU-LWNA-33	Increasing P solubilization	[23]
	*Herbaspirillum* sp. H18 and ZA15, *Burkholderia vietaminensis* AR114	Increasing P solubilization and N use efficiency	[133]
	*Enterobacter cloacae* RCA25, *Klebsiella variicola* RCA26, *Herbaspirillum seropedicae* z67, *Sinorhizobium fredii* NGR234	IAA production, improving N fixation	[134]
	*Paenibacillus* spp.	Catalyzing N_2_-fixing	[135]
	*Pseudomonas* sp. S10-3	Increasing K solubilization	[136]
	*Burkholderia* spp.	Increasing K solubilization	[137]
	*Pantoea agglomerans*, *Rahnella aquatilis*, *Pseudomonas orientalis*	Increasing K solubilization, IAA production	[138]
	*Providencia rettgeri* TPM23	Increasing available N, P and K contents	[139]
	*Enterobacter cloacae* HG-1	Enhancing N fixation, producing iron carriers, ACC deaminase and plant hormones	[140]
	Rhizobium strains NSFBR-12, NSFBR-15	Enhancing N fixation	[141]
	*Azospirillum brasilense*	Improving fertilizer-N recovery	[142]
Pesticide residues	*Microbacterium* sp. D-2	Dicofol degradation	[143]
	*Acinetobacter baylyi* GFJ2	Chloroanilines degradation	[144]
	*Pseudomonas* sp.	DDT degradation	[145]
	*Pseudomonas putida*, *Acinetobacter rhizosphaerae*	Hydrolysis of organophosphate and carbamate pesticides	[146]
	*Chryseobacterium* sp. Y16C	Glyphosate degradation	[147]
	*Arthrobacter* sp. HB-5	Atrazine degradation	[148]
	*Trichoderma atroviride* T23	Dichlorvos degradation	[149]
	*Alcaligenes faecalis* NBRI OSS2-5	Chlorpyrifos degradation	[150]
	*Bacillus pumilus* W1	Organophosphate degradation	[151]
	*Pseudomonas nitroreducens* AR-3	Chlorpyrifos degradation	[152]
	*Pseudomonas putida* ATCC 49451	Chlorophenols degradation	[153]
	*Bacillus aryabhattai* VITNNDJ5	Monocrotophos degradation	[154]
	*Sphingomonas* sp. AK1	Isoproturon degradation	[155]
	*Bacillus* spp.	Diuron degradation	[156]
	*Catellibacterium* sp. CC-5	Cypermethrin degradation	[157]
	*Xanthomonas axonopodis*, *Aspergillus niger*	Alachlor degradation	[158]
	*Bacillus cereus* Y1	Deltamethrin degradation	[159]
Excessive heavy metals	*Bacillus thuringiensis* WS3	As removal	[160]
	*Pseudomonas putida* UW4	PAH and Pb removal	[161]
	*Kocuria rhizophila*	Promoting phytoremediation of Cd, Cr, Cu and Ni-contaminated soil	[162]
	*Bacillus cereus* HM5, *Bacillus* *thuringiensis* HM7	Improving Mn phytoremediation	[163]
	*Pseudomonas lurida* EOO26	Improving Cu phytoremediation	[164]
	*Bacillus subtilus* MAI3	Chromium (VI) reduction and Cr (III) immobilization	[165]
	*Trichoderma harzianum*, *Bacillus subtilis*	Cd bioavailability reduction	[166]
	*Pantoea conspicua* MT5, *Aspergillus* *niger* CRS3	Reduction and detoxification of Cr (Ⅵ)	[167]
Organic pollutants	*Hyphomicrobium* sp. GHH	17α-ethinyestradiol removal	[168]
	*Klebsiella pneumonia* PL1	Pyrene and benzo(a)pyrene removal	[169]
	*Pseudomonas mendocina*, *Brevundimonas* *olei*, *Serratia marcescens*	Creosote PAH degradation	[170]
	*Psuedomonas* sp. USTB-RU	Phenanthrene degradation	[171]
	*Sphingobium yanoikuyae* B1	PAH degradation	[172]
	*Pseudomonas* sp. SDR4, *Mortierella* *alpina* JDR7	PAH degradation	[173]
	*Mycobacterium* spp.	Pyrene degradation	[174]
	*Ralstonia* sp. U2	Naphthalene degradation	[175]
	*Pseudomonas aeruginosa* PSA5, *Rhodococcus* sp. NJ2	Benzo(a)pyrene degradation	[176]
	*Pseudomonas oleovorans* DT4	Tetrahydrofuran, benzene, toluene, ethylbenzene and xylene degradation	[177]
	*Raoultella* sp. XY-1, *Pandoraea* sp. XY-2	Tetracycline degradation	[178]

**Table 2:** Role of beneficial microorganisms on growth promotion and quality improvement of medicinal plants.

Medicinal Plants	Strains/Microbes	Role	Reference
*Panax ginseng*	*Paenibacillus polymyxa*	Simultaneous degradation of fluazinam, BHC, PCNB, chlorpyrifos and DDT in roots, stems and leaves	[26]
	*Bacillus subtilis* 50-1	Replanting mortality alleviation	[229]
	*Bacillus amyloliquefaciens* SW-34	Grey mold suppression	[230]
	*Bacillus amyloliquefaciens* HK34	Foliar blight and root rot control	[231]
	*Pseudomonas aeruginosa* D4 and *Bacillus Stratosphericus* FW3	Root rot fungal pathogen control	[232]
	*Rhizobium panacihumi* DCY116^T^	Al resistance enhancement	[233]
	*Sphingobacterium* sp. PG-1	Toxic diisobutyl phthalate degradation, replanting issue alleviation, growth promotion	[234]
*P. quinquefolius*	*Rhizoglomus irregulare*, *Funneliformis mosseae* and *F. caledonium*	Soil-borne pathogen control, nutrient acquisition improvement, continuous cropping obstacle mitigation	[235]
*Glycyrrhiza glabra*	*Funneliformis mosseae*	Salt stress alleviation, P and K-concentration increase, glycyrrhizin accumulation	[236]
*G. uralensis*	*Glomus mosseae* and *G. veriforme*	Growth promotion, P-acquisition improvement, glycyrrhizin accumulation	[237]
	*Rhizophagus irregularis*	Compensation for the loss of indigenous microbial communities, growth and secondary metabolism promotion	[238]
	*Acrocalymma vagum*	Biomass increase under drought stress, glycyrrhizic acid and glycyrrhizin accumulation	[239]
*Salvia miltiorrhiza*	*Bacillus cereus*	Hairy root growth promotion, tanshinone production increase	[240]
	*Bacillus amyloliquefaciens*, *B. licheniformis* and *Actinomyces bovis*	Cd-uptake reduction, total tanshinones accumulation	[241]
*S. officinalis*	*Glomus intraradices*	Essential oil yield and quality improvement	[242]
*Artemisia annua*	*Colletotrichum* sp.	Artemisinin production stimulation	[243]
	*Rizophagus irregularis*	Artemisinin content and essential oil yield increase	[244]
	*Glomus mosseae* and *Bacillus subtilis* Daz26	Growth, biomass yield, and artemisinin content enhancement	[245]
*Sophora flavescens*	Actinobacteria and Chloroflexi	Matrine and oxymatrine accumulation	[246]
	*Bradyrhizobium arachidis* CCBAU 051107^T^	Oxymatrine and matrine content enhancement	[247]
*Pistacia vera*	*Staphylococcus sciuri*, *Zobellella denitrificans* and *Arthrobacter endophyticus*	Yield increase, photosynthesis promotion, water absorption promotion, performance improvement under salinity and drought stresses	[248]
*Bacopa monnieri*	*Pseudomonas plecoglossicida* KM233646, *Acinetobacter calcoaceticus* KM233647, *Bacillus flexus* KM233648 and *B. safensis* KM233652	Growth promotion, bacoside A yield increase, saline soil reclamation	[249]
*Echinacea purpurea*	*Pseudomonas fluorescens*, AMF	Nutrient concentration increase, water absorption promotion, growth improvement, drought stress alleviation	[250]
*Trigonella foenum-graecum*	*Bacillus subtilis* LDR2	Nodulation and AMF colonization enhancement, nutrient uptake improvement, growth promotion, drought-stress resistance enhancement	[251]
	*Azotobacter chroococcum* and *Peudomoance fluorescence*	Yield and trigonelline production enhancement under deficit irrigation	[252]
*Foeniculum vulgare*	*Bacillus subtilis* PSB-1 and PSB-36	Seed yield and essential oil content increase, P-availability enhancement	[253]
*Rheum palmatum*	*Bacillus amyloliquefaciens* EZ99	Increase of root fresh weight, active components content and K-availability	[254]
*Naravelia zeylanica*	*Achromobacter xylosoxidans* AUM54	Growth promotion, survivability and stress tolerance increase	[255]
*Astragalus mongholicus*	*Stenotrophomonas*, *Phyllobacterium* and *Inquilinus*	Bioactive ingredients accumulation	[256]
*Polygonum cuspidatum*	*Bacteroides*, *Acinetobacter*, *Erysipelatoclostridium* and *Achromobacter*	Resveratrol accumulation	[257]
*Chrysanthemum morifolium*	*Funneliformis mosseae* and *Diversispora versiformis*	N-uptake enhancement, growth promotion under salt stress	[258]
*Papaver somniferum*	*Pseudomonas putida*	Growth and yield improvement, downy mildew tolerance enhancement	[259]
*Zingiber officinale*	*Bacillus*, *Pseudomonas*, *Arthrobacter* and *Serratia*	Al-toxicity and bacterial wilt alleviation	[260]
*Andrographis paniculata*	*Bacillus* sp. CIMAP-A7	Atrazine-induced toxicity amelioration, oxidative stress amelioration	[261]
	*Azotobacter chrococcum*, *Bacillus* *megaterium*, *Pseudomonas monteilii*, *Glomus intraradices*	Soil dehydrogenase, alkaline and acidic phosphatase activity improvement, growth promotion, pharmacological quality enhancement	[262]
*Commiphora leptophloeos*	*Gigaspora albida* and *Claroideoglomus etunicatum*	Total foliar phenols and tannins increase	[263]
*Ocimum basilicum*	*Glomus caledonium* BEG 162 and *G. mosseae* NBR 1–2	Rosmarinic and caffeic acids production enhancement	[264]
*Atractylodes lancea*	*Pseudomonas fluorescens* ALEB7B	Medicinal sesquiterpenoid accumulation, photosynthesis enhancement	[265]
*Hypericum perforatum*	*Stenotrophomonas maltophilia* N5.18	Hypericin and pseudohypericin increase	[266]
	*Rhizophagus intraradices*, *Funneliformis constrictum, F. geosporum* and *F. mosseae*	Photosynthetic activity stimulation, secondary metabolites production, hypericin and pseudohypericin concentration enhancement	[267]
*Hyoscyamus niger*	*Pseudomonas putida* and *P. fluorescens*	Growth promotion under water deficit stress, alkaloid production increase	[268]
*Crocus sativus*	*Rhizophagus intraradicse*, *Funneliformis mosseae*, *Rhizophagus irregutaris* and *Glomus caledonium*	Photosynthesis promotion, increase of flower number, leaf dry weight and area and yield	[269]
*Mentha arvensis*	*Exiguobacterium oxidotoleran,* and *Glomus fasciculatum*	Growth promotion in salt-stressed soil, essential oil yield increase	[270]
	*Trichoderma harzianum* and *Brevibacterium halotolerans*	Increase in plant growth, oil content, leaf-stem ratio, photosynthetic pigments and nutrient uptake	[271]

## References

[1] Wang W.Y., Zhou H., Wang Y.F., Sang B.S., Liu L. (2021). Current policies and measures on the development of traditional Chinese medicine in China. Pharmacol. Res..

[2] Astutik S., Pretzsch J., Kimengsi J.N. (2019). Asian medicinal plants’ production and utilization potentials: A review. Sustainability.

[3] Yuan Y., Tang X., Jia Z., Li C., Ma J., Zhang J. (2020). The effects of ecological factors on the main medicinal components of *Dendrobium officinale* under different cultivation modes. Forests.

[4] Zhang M., Jiang D., Yang M., Ma T., Ding F., Hao M., Chen Y., Zhang C., Zhang X., Li M. (2021). Influence of the environment on the distribution and quality of *Gentiana dahurica* Fisch. Front. Plant Sci..

[5] Zeng Z., Mou D., Luo L., Zhong W., Duan L., Zou X. (2021). Different cultivation environments affect the yield, bacterial community and metabolites of *Cordyceps cicadae*. Front. Microbiol..

[6] Dong Z., Rao M.P.N., Liao T., Li L., Liu Y., Xiao M., Mohamad O.A.A., Tian Y., Li W. (2021). Diversity and function of rhizosphere microorganisms between wild and cultivated medicinal plant *Glycyrrhiza uralensis* Fisch under different soil conditions. Arch. Microbiol..

[7] Lang T., Pan L., Liu B., Guo T., Hou X. (2020). Vegetation characteristics and response to the soil properties of three medicinal plant communities in Altay Prefecture, China. Sustainability.

[8] Xin A., Li X., Jin H., Yang X., Zhao R., Liu J., Qin B. (2019). The accumulation of reactive oxygen species in root tips caused by autotoxic allelochemicals-A significant factor for replant problem of *Angelica sinensis* (Oliv.) Diels. Ind. Crop. Prod..

[9] Vasickova J., Hvezdova M., Kosubova P., Hofman J. (2019). Ecological risk assessment of pesticide residues in arable soils of the Czech Republic. Chemosphere.

[10] Zhang Y., Zheng Y., Xia P., Xun L., Liang Z. (2019). Impact of continuous *Panax notoginseng* plantation on soil microbial and biochemical properties. Sci. Rep..

[11] Pang Z., Mao X., Xia Y., Xiao J., Wang X., Xu P., Liu G. (2022). Multiomics reveals the effect of root rot on Polygonati rhizome and identifies pathogens and biocontrol strain. Microbiol. Spectr..

[12] Sharma P., Pandey A.K., Kim S., Singh S.P., Chaturvedi P., Varjani S. (2021). Critical review on microbial community during in-situ bioremediation of heavy metals from industrial wastewater. Environ. Technol. Innov..

[13] Geng Y., Jiang L., Jiang H., Wang L., Peng Y., Wang C., Shi X., Gu J., Wang Y., Zhu J. (2019). Assessment of heavy metals, fungicide quintozene and its hazardous impurity residues in medical *Panax notoginseng* (Burk) F.H. Chen root. Biomed. Chromatogr..

[14] The Ministry of Environmental Protection, The Ministry of Land and Resources (2014). Report on the National General Survey of Soil Contamination. https://www.mee.gov.cn/gkml/sthjbgw/qt/201404/W020140417558995804588.pdf.

[15] Ara T., Nisa W.U., Aziz R., Rafiq M.T., Gill R.A., Hayat M.T., Afridi U. (2021). Health risk assessment of hexachlorocyclohexane in soil, water and plants in the agricultural area of Potohar region, Punjab, Pakistan. Environ. Geochem. Health.

[16] Fu Y., Dou X., Lu Q., Qin J., Luo J., Yang M. (2020). Comprehensive assessment for the residual characteristics and degradation kinetics of pesticides in *Panax notoginseng* and planting soil. Sci. Total Environ..

[17] Swamy M.K., Nalina N., Nalina D., Akhtar M.S., Purushotham B., Akhtar M. (2019). Heavy metal stress and tolerance in plants mediated by rhizospheric microbes. Salt Stress, Microbes, and Plant Interactions: Causes and Solution.

[18] Upton R.N., Bach E.M., Hofmockel K.S. (2019). Spatio-temporal microbial community dynamics within soil aggregates. Soil Biol. Biochem..

[19] Kui L., Chen B., Chen J., Sharifi R., Dong Y., Zhang Z., Miao J. (2021). A comparative analysis on the structure and function of the *Panax notoginseng* rhizosphere microbiome. Front. Microbiol..

[20] Genre A., Lanfranco L., Perotto S., Bonfante P. (2020). Unique and common traits in mycorrhizal symbioses. Nat. Rev. Microbiol..

[21] Vejan P., Abdullah R., Khadiran T., Ismail S., Nasrulhaq B.A. (2016). Role of plant growth promoting rhizobacteria in agricultural sustainability—A review. Molecules.

[22] Khan I., Aftab M., Shakir S., Ali M., Qayyum S., Rehman M.U., Haleem K.S., Touseef I. (2019). Mycoremediation of heavy metal (Cd and Cr)-polluted soil through indigenous metallotolerant fungal isolates. Environ. Monit. Assess..

[23] Kour D., Rana K.L., Sheikh I., Kumar V., Yadav A.N., Dhaliwal H.S., Saxena A.K. (2019). Alleviation of drought stress and plant growth promotion by *Pseudomonas libanensis* EU-LWNA-33, a drought-adaptive phosphorus-solubilizing bacterium. Proc. Nat. Acad. Sci. India Sect. B Biol. Sci..

[24] Chaudhary D.K., Kim J. (2016). *Novosphingobium naphthae* sp. nov., from oil-contaminated soil. Int. J. Syst. Evol. Microbiol..

[25] Kalaimurugan D., Balamuralikrishnan B., Durairaj K., Vasudhevan P., Shivakumar M.S., Kaul T., Chang S.W., Ravindran B., Venkatesan S. (2019). Isolation and characterization of heavy-metal-resistant bacteria and their applications in environmental bioremediation. Int. J. Environ. Sci. Technol..

[26] Zhang X., Gao Y., Zang P., Zhao Y., He Z., Zhu H., Song S., Zhang L. (2019). Study on the simultaneous degradation of five pesticides by *Paenibacillus polymyxa* from *Panax ginseng* and the characteristics of their products. Ecotoxicol. Environ. Saf..

[27] Alawiye T., Babalola O. (2021). Metagenomic insight into the community structure and functional genes in the sunflower rhizosphere microbiome. Agriculture.

[28] Brunel C., Pouteau R., Dawson W., Pester M., Ramirez K.S., Van Kleunen M. (2020). Towards unraveling macroecological patterns in rhizosphere microbiomes. Trends Plant Sci..

[29] Saleem M., Hu J., Jousset A. (2019). More than the sum of its parts: Microbiome biodiversity as a driver of plant growth and soil health. Annu. Rev. Ecol. Evol. Syst..

[30] Cao P., Wang G., Wei X., Chen S., Han J. (2021). How to improve CHMs quality: Enlighten from CHMs ecological cultivation. Chin. Herb. Med..

[31] Haider F.U., Cai L., Coulter J.A., Cheema S.A., Wu J., Zhang R., Ma W., Farooq M. (2021). Cadmium toxicity in plants: Impacts and remediation strategies. Ecotoxicol. Environ. Saf..

[32] Li Y., Dai S., Wang B., Jiang Y., Ma Y., Pan L., Wu K., Huang X., Zhang J., Cai Z. (2020). Autotoxic ginsenoside disrupts soil fungal microbiomes by stimulating potentially pathogenic microbes. Appl. Environ. Microbiol..

[33] Li B., Zhang Q., Chen Y., Su Y., Sun S., Chen G. (2021). Different crop rotation systems change the rhizosphere bacterial community structure of *Astragalus membranaceus* (Fisch) Bge. var. mongholicus (Bge.) Hsiao. Appl. Soil Ecol..

[34] Pang Z., Tayyab M., Kong C., Liu Q., Liu Y., Hu C., Huang J., Weng P., Islam W., Lin W. (2021). Continuous sugarcane planting negatively impacts soil microbial community structure, soil fertility, and sugarcane agronomic parameters. Microorganisms.

[35] Gao Z., Hu Y., Han M., Xu J., Wang X., Liu L., Tang Z., Jiao W., Jin R., Liu M. (2021). Effects of continuous cropping of sweet potatoes on the bacterial community structure in rhizospheric soil. BMC Microbiol..

[36] Pang Z., Dong F., Liu Q., Lin W., Hu C., Yuan Z. (2021). Soil metagenomics reveals effects of continuous sugarcane cropping on the structure and functional pathway of rhizospheric microbial community. Front. Microbiol..

[37] Xia F., Wang L., Chen J., Fu M., Wang G., Yan Y., Cui L. (2021). Variations of microbial community in *Aconitum carmichaeli* Debx. rhizosphere soilin a short-term continuous cropping system. J. Microbiol..

[38] Liu H., Niu M., Zhu S., Zhang F., Liu Q., Liu Y., Liu R., Zhang Y. (2020). Effect study of continuous monoculture on the quality of *Salvia miltiorrhiza* Bge Roots. Biomed. Res. Int..

[39] Zhang Y., Guo R., Li S., Chen Y., Li Z., He P., Huang X., Huang K. (2021). Effects of continuous cropping on soil, senescence, and yield of *Tartary buckwheat*. Agron. J..

[40] Guo K., He X., Yan Z., Li X., Ren X., Pan L., Qin B. (2016). Allelochemicals from the rhizosphere soil of cultivated *Astragalus hoantchy*. J. Agric. Food Chem..

[41] Zhang W., Lu L.-Y., Hu L.-Y., Cao W., Sun K., Sun Q.-B., Siddikee A., Shi R.-H., Dai C.-C. (2018). Evidence for the involvement of auxin, ethylene and ROS signaling during primary root inhibition of Arabidopsis by the allelochemical benzoic acid. Plant Cell Physiol..

[42] Tan G., Liu Y., Peng S., Yin H., Meng D., Tao J., Gu Y., Li J., Yang S., Xiao N. (2021). Soil potentials to resist continuous cropping obstacle: Three field cases. Environ. Res..

[43] Ren X., Yan Z., He X., Li X., Qin B. (2017). Allelochemicals from rhizosphere soils of *Glycyrrhiza uralensis* Fisch: Discovery of the autotoxic compounds of a traditional herbal medicine. Ind. Crop. Prod..

[44] Li X., Lewis E.E., Liu Q., Li H., Bai C., Wang Y. (2016). Effects of long-term continuous cropping on soil nematode community and soil condition associated with replant problem in strawberry habitat. Sci. Rep..

[45] Liu S., Wang Z., Niu J., Dang K., Zhang S., Wang S., Wang Z. (2021). Changes in physicochemical properties, enzymatic activities, and the microbial community of soil significantly influence the continuous cropping of *Panax quinquefolius* L. (American ginseng). Plant Soil.

[46] Zhang B., Li X., Wang F., Li M., Zhang J., Gu L., Zhang L., Tu W., Zhang Z. (2016). Assaying the potential autotoxins and microbial community associated with *Rehmannia glutinosa* replant problems based on its ‘autotoxic circle’. Plant Soil.

[47] Lei H., Liu A., Hou Q., Zhao Q., Guo J., Wang Z. (2020). Diversity patterns of soil microbial communities in the *Sophora flavescens* rhizosphere in response to continuous monocropping. BMC Microbiol..

[48] Wei X., Wang X., Cao P., Gao Z., Chen A.J., Han J. (2020). Microbial community changes in the rhizosphere soil of healthy and rusty *Panax ginseng* and discovery of pivotal fungal genera associated with rusty roots. Biomed Res. Int..

[49] Zhu B., Wu J., Ji Q., Wu W., Dong S., Yu J., Zhang Q., Qin L. (2020). Diversity of rhizosphere and endophytic fungi in *Atractylodes macrocephala* during continuous cropping. PeerJ.

[50] Gao Z., Han M., Hu Y., Li Z., Liu C., Wang X., Tian Q., Jiao W., Hu J., Liu L. (2019). Effects of continuous cropping of sweet potato on the fungal community structure in rhizospheric soil. Front. Microbiol..

[51] Kulmatov R., Mirzaev J., Abuduwaili J., Karimov B. (2020). Challenges for the sustainable use of water and land resources under a changing climate and increasing salinization in the Jizzakh irrigation zone of Uzbekistan. J. Arid Land.

[52] Zhang Z., Sun D., Tang Y., Zhu R., Li X., Gruda N., Dong J., Duan Z. (2021). Plastic shed soil salinity in China: Current status and next steps. J. Clean. Prod..

[53] Wu R., Sun H., Xue J., Yan D., Liu Y., Gui D., Wang X., Yang J. (2020). Acceleration of soil salinity accumulation and soil degradation due to greenhouse cultivation: A survey of farmers’ practices in China. Environ. Monit. Assess..

[54] Figueiredo P.G., Bicudo S.J., Chen S., Fernandes A.M., Tanamati F.Y., Djabou-Fondjo A.S.M. (2017). Effects of tillage options on soil physical properties and cassava-dry-matter partitioning. Field Crop. Res..

[55] Rengasamy P. (2006). World salinization with emphasis on Australia. J. Exp. Bot..

[56] Etesami H., Beattie G.A. (2018). Mining halophytes for plant growth-promoting halotolerant bacteria to enhance the salinity tolerance of non-halophytic crops. Front. Microbiol..

[57] Bian L., Wang J., Liu J., Han B. (2021). Spatiotemporal changes of soil salinization in the Yellow River Delta of China from 2015 to 2019. Sustainability.

[58] Huang J., Hartemink A.E. (2020). Soil and environmental issues in sandy soils. Earth-Sci. Rev..

[59] Buvaneshwari S., Riotte J., Sekhar M., Sharma A.K., Helliwell R., Kumar M., Braun J.J., Ruiz L. (2020). Potash fertilizer promotes incipient salinization in groundwater irrigated semi-arid agriculture. Sci. Rep..

[60] Chen Y.P., Rekha P.D., Arun A.B., Shen F.T., Lai W.A., Young C.C. (2006). Phosphate solubilizing bacteria from subtropical soil and their tricalcium phosphate solubilizing abilities. Appl. Soil Ecol..

[61] Elhanafi L., Houhou M., Rais C., Mansouri I., Elghadraoui L., Greche H. (2019). Impact of excessive nitrogen fertilization on the biochemical quality, phenolic compounds, and antioxidant power of *Sesamum indicum* L Seeds. J. Food Qual..

[62] Ameixa O.M.C.C., Marques B., Fernandes V.S., Soares A.M.V.M., Calado R., Lillebø A.I. (2016). Dimorphic seeds of *Salicornia ramosissima* display contrasting germination responses under different salinities. Ecol. Eng..

[63] Mahajan M., Sharma S., Kumar P., Pal P.K. (2020). Foliar application of KNO_3_ modulates the biomass yield, nutrient uptake and accumulation of secondary metabolites of *Stevia rebaudiana* under saline conditions. Ind. Crop. Prod..

[64] Mohanavelu A., Naganna S.R., Al-Ansari N. (2021). Irrigation induced salinity and sodicity hazards on soil and groundwater: An overview of its causes, impacts and mitigation strategies. Agriculture.

[65] Ashraf M., Foolad M.R. (2007). Roles of glycine betaine and proline in improving plant abiotic stress resistance. Environ. Exp. Bot..

[66] Mansour M.M.F. (1998). Protection of plasma membrane of onion epidermal cells by glycinebetaine and proline against NaCl stress. Plant Physiol. Biochem..

[67] Ketchum R.E.B., Warren R.S., Klima L.J., Lopez-Gutiérrez F., Nabors M.W. (1991). The mechanism and regulation of proline accumulation in suspension cell cultures of the halophytic grass *Distichlis spicata* L.. J. Plant Physiol..

[68] Kubotera H. (2020). Analysis of problems in certain soils of the Kyushu Okinawa region for suitable management. Soil Sci. Plant Nutr..

[69] Wahlström E.M., Kristensen H.L., Thomsen I.K., Labouriau R., Pulido-Moncada M., Nielsen J.A., Munkholm L.J. (2021). Subsoil compaction effect on spatio-temporal root growth, reuse of biopores and crop yield of spring barley. Eur. J. Agron..

[70] Gaab T. (2014). Effect of soil compaction and N fertilization on soil pore characteristics and physical quality of sandy loam soil under red clover/grass sward. Soil Tillage Res..

[71] Pandey B.K., Huang G., Bhosale R., Hartman S., Sturrock C.J., Jose L., Martin O.C., Karady M., Voesenek L.A.C.J., Ljung K. (2021). Plant roots sense soil compaction through restricted ethylene diffusion. Science.

[72] Zhang H., Voroney R.P., Price G.W., White A.J. (2017). Sulfur-enriched biochar as a potential soil amendment and fertiliser. Soil Res..

[73] Iturri L.A., Buschiazzo D.E. (2016). Light acidification in N-fertilized loess soils along a climosequence affected chemical and mineralogical properties in the short term. Catena.

[74] Wu Z., Sun X., Sun Y., Yan J., Zhao Y., Chen J. (2022). Soil acidification and factors controlling topsoil pH shift of cropland in central China from 2008 to 2018. Geoderma.

[75] Li Y., Sun J., Tian D., Wang J., Ha D., Qu Y., Jing G., Niu S. (2018). Soil acid cations induced reduction in soil respiration under nitrogen enrichment and soil acidification. Sci. Total Environ..

[76] Cecchini G., Andreetta A., Marchetto A., Carnicelli S. (2019). Atmospheric deposition control of soil acidification in central Italy. Catena.

[77] Huang J., Zhou K., Zhang W., Liu J., Ding X., Cai X., Mo J. (2019). Sulfur deposition still contributes to forest soil acidification in the Pearl River Delta, South China, despite the control of sulfur dioxide emission since 2001. Environ. Sci. Pollut. Res. Int..

[78] Zhang L., Qiu Y., Cheng L., Wang Y., Liu L., Tu C., Bowman D.C., Burkey K.O., Bian X., Zhang W. (2018). Atmospheric CO_2_ enrichment and reactive nitrogen inputs interactively stimulate soil cation losses and acidification. Environ. Sci. Technol..

[79] Raza S., Miao N., Wang P., Ju X., Chen Z., Zhou J., Kuzyakov Y., Na M. (2020). Dramatic loss of inorganic carbon by nitrogen-induced soil acidification in Chinese croplands. Glob. Chang. Biol..

[80] Cai Z., Wang B., Xu M., Zhang H., He X., Zhang L., Gao S. (2014). Intensified soil acidification from chemical N fertilization and prevention by manure in an 18-year field experiment in the red soil of southern China. J. Soils Sediments.

[81] Hao T., Zhu Q., Zeng M., Shen J., Shi X., Liu X., Zhang F., de Vries W. (2020). Impacts of nitrogen fertilizer type and application rate on soil acidification rate under a wheat-maize double cropping system. J. Environ. Manag..

[82] Hao T., Zhu Q., Zeng M., Shen J., Shi X., Liu X., Zhang F., de Vries W. (2019). Quantification of the contribution of nitrogen fertilization and crop harvesting to soil acidification in a wheat-maize double cropping system. Plant Soil.

[83] Zhao Q., Xiong W., Xing Y., Sun Y., Lin X., Dong Y. (2018). Long-term coffee monoculture alters soil chemical properties and microbial communities. Sci. Rep..

[84] Coventry D.R., Slattery W.J. (1991). Acidification of soil associated with lupins grown in a crop rotation in north-eastern Victoria. Aust. J. Agric. Res..

[85] Zhang Y., He X., Zhao J., Zhang Y., Shi X. (2016). Soil acidification under long-term tobacco plantation results in alterations of mineralogical properties in an Alisol. Arch. Agron. Soil Sci..

[86] Yu Y., Yang J., Zeng S., Wu D., Jacobs D.F., Sloan J.L. (2016). Soil pH, organic matter, and nutrient content change with the continuous cropping of *Cunninghamia lanceolata* plantations in South China. J. Soils Sediments.

[87] Zhang Y., He X., Liang H., Zhao J., Zhang Y., Xu C., Shi X. (2016). Long-term tobacco plantation induces soil acidification and soil base cation loss. Environ. Sci. Pollut. Res. Int..

[88] Golez N.V., Kyuma K. (1997). Influence of pyrite oxidation and soil acidification on some essential nutrient elements. Aquac. Eng..

[89] Kang Y., Zhu S., Li G., Jiang Y., Teng W., Luo M., Wei J., Cao F., Wang Z., Huang J. (2020). Phosphorus applied to the root half without Al^3+^ exposure can alleviate Al toxicity on the other root half of the same eucalyptus seedling. J. Plant Nutr..

[90] Zarif N., Khan A., Wang Q. (2020). Linking soil acidity to P fractions and exchangeable base cations under increased N and P fertilization of mono and mixed plantations in Northeast China. Forests.

[91] Babourina O., Hawkins B., Lew R.R., Newman I., Shabala S. (2001). K^+^ transport by Arabidopsis root hairs at low pH. Funct. Plant Biol..

[92] Chen L., Guo L., Zhou Q., Liu M., Zhan S., Pan X., Zeng Y. (2020). Response of soil fertility and Cu and Cd availability to biochar application on paddy soils with different acidification levels. Biomass Conv. Bioref..

[93] Zhang F., Jin Q., Peng H., Zhu T. (2021). Soil acidification in moso bamboo (*Phyllostachys edulis*) forests and changes of soil metal ions (Cu, Pb) concentration. Arch. Agron. Soil Sci..

[94] Liu C., Liang M., Nie Y., Tang J., Siddique K.H.M. (2019). The conversion of tropical forests to rubber plantations accelerates soil acidification and changes the distribution of soil metal ions in topsoil layers. Sci. Total Environ..

[95] Zhou X., Khashi U.R.M., Liu J., Wu F. (2021). Soil acidification mediates changes in soil bacterial community assembly processes in response to agricultural intensification. Environ. Microbiol..

[96] Xing W., Lu X., Ying J., Lan Z., Chen D., Bai Y. (2022). Disentangling the effects of nitrogen availability and soil acidification on microbial taxa and soil carbon dynamics in natural grasslands. Soil Biol. Biochem..

[97] Chen Z., Maltz M.R., Zhang Y., O’Brien B.J., Neff M., Wang Y., Cao J. (2021). Plantations of *Cinnamomum camphora* (Linn) Presl with distinct soil bacterial communities mitigate soil acidity within polluted locations in Southwest China. Forests.

[98] Muneer M.A., Hou W., Li J., Huang X., ur Rehman Kayani M., Cai Y., Yang W., Wu L., Ji B., Zheng C. (2022). Soil pH: A key edaphic factor regulating distribution and functions of bacterial community along vertical soil profiles in red soil of pomelo orchard. BMC Microbiol..

[99] Li J., Zheng T., Liu C. (2021). Soil acidification enhancing the growth and metabolism inhibition of PFOS and Cr(VI) to bacteria involving oxidative stress and cell permeability. Environ. Pollut..

[100] Grube A., Donaldson D., Kiely T., Wu L. (2011). Pesticides Industry Sales and Usage: 2006 and 2007 Market Estimates. https://www.epa.gov/sites/default/files/2015-10/documents/market_estimates2007.pdf.

[101] Kumar S., Kaushik G., Dar M.A., Nimesh S., Lopez-Chuken U.J., Villarreal-Chiu J.F. (2018). Microbial degradation of organophosphate pesticides; A review. Pedosphere.

[102] Kaur I., Gaur V.K., Regar R.K., Roy A., Srivastava P.K., Gaur R., Manickam N., Barik S.K. (2021). Plants exert beneficial influence on soil microbiome in a HCH contaminated soil revealing advantage of microbe-assisted plant-based HCH remediation of a dumpsite. Chemosphere.

[103] Ahmad K.S., Gul P., Gul M.M. (2020). Efficient fungal and bacterial facilitated remediation of thiencarbazone methyl in the environment. Environ. Res..

[104] Wang L., Wang J., Zhu L., Wang J. (2018). Toxic effects of oxytetracycline and copper, separately or combined, on soil microbial biomasses. Environ. Geochem. Health.

[105] Jampani M., Huelsmann S., Liedl R., Sonkamble S., Ahmed S., Amerasinghe P. (2018). Spatio-temporal distribution and chemical characterization of groundwater quality of a wastewater irrigated system: A case study. Sci. Total Environ..

[106] Sikka R., Nayyar V., Sidhu S.S. (2009). Monitoring of Cd pollution in soils and plants irrigated with untreated sewage water in some industrialized cities of Punjab, India. Environ. Monit. Assess..

[107] Shaheen S.M., Shams M.S., Khalifa M.R., El-Dali M.A., Rinklebe J. (2017). Various soil amendments and environmental wastes affect the (im) mobilization and phytoavailability of potentially toxic elements in a sewage effluent irrigated sandy soil. Ecotoxicol. Environ. Saf..

[108] Qin G., Niu Z., Yu J., Li Z., Ma J., Xiang P. (2021). Soil heavy metal pollution and food safety in China: Effects, sources and removing technology. Chemosphere.

[109] Jin Y., Luan Y., Ning Y., Wang L. (2018). Effects and mechanisms of microbial remediation of heavy metals in soil: A critical review. Appl. Sci..

[110] Taha S.M., Gadalla S.A. (2017). Development of an efficient method for multi residue analysis of 160 pesticides in herbal plant by ethyl acetate hexane mixture with direct injection to GC-MS/MS. Talanta.

[111] Durgnat J.M., Heuser J., Andrey D., Perrin C. (2005). Quality and safety assessment of ginseng extracts by determination of the contents of pesticides and metals. Food Addit. Contam..

[112] Kumar N., Kulsoom M., Shukla V., Kumar D., Priyanka, Kumar S., Tiwari J., Dwivedi N. (2018). Profiling of heavy metal and pesticide residues in medicinal plants. Environ. Sci. Pollut. Res. Int..

[113] Harris E.S., Cao S., Littlefield B.A., Craycroft J.A., Scholten R., Kaptchuk T., Fu Y., Wang W., Liu Y., Chen H. (2011). Heavy metal and pesticide content in commonly prescribed individual raw Chinese herbal medicines. Sci. Total Environ..

[114] Maitlo A.A., Jatoi W.B., Memon A.F., Soomro A.H., Bhayo M.S. (2021). Assessment of Zinc, Lead, Chromium, and Cobalt in commonly consumed herbal medicines in Sindh, Pakistan. Biol. Trace Elem. Res..

[115] Singh S., Singh A., Bashri G., Prasad S.M. (2016). Impact of Cd stress on cellular functioning and its amelioration by phytohormones: An overview on regulatory network. Plant Growth Regul..

[116] Scoccianti V., Crinelli R., Tirillini B., Mancinelli V., Speranza A. (2006). Uptake and toxicity of Cr(III) in celery seedlings. Chemosphere.

[117] Ahsan M., Younis A., Jaskani M.J., Tufail A., Riaz A., Schwinghamer T., Tariq U., Nawaz F. (2018). Heavy metal accumulation imparts structural differences in fragrant *Rosa* species irrigated with marginal quality water. Ecotoxicol. Environ. Saf..

[118] Liu L., Li S., Guo J., Li N., Jiang M., Li X. (2022). Low temperature tolerance is depressed in wild-type and abscisic acid-deficient mutant barley grown in Cd-contaminated soil. J. Hazard. Mater..

[119] Wang Q., Zhang J., Zhao B., Xin X., Zhang C., Zhang H. (2014). The influence of long-term fertilization on cadmium (Cd) accumulation in soil and its uptake by crops. Environ. Sci. Pollut. Res. Int..

[120] Park H.J., Kim S.U., Jung K.Y., Lee S., Choi Y.D., Owens V.N., Kumar S., Yun S.W., Hong C.O. (2021). Cadmium phytoavailability from 1976 through 2016: Changes in soil amended with phosphate fertilizer and compost. Sci. Total Environ..

[121] Pizzol M., Smart J.C.R., Thomsen M. (2014). External costs of cadmium emissions to soil: A drawback of phosphorus fertilizers. J. Clean. Prod..

[122] Zhang S., Yao H., Lu Y., Yu X., Wang J., Sun S., Liu M., Li D., Li Y.-F., Zhang D. (2017). Uptake and translocation of polycyclic aromatic hydrocarbons (PAHs) and heavy metals by maize from soil irrigated with wastewater. Sci. Rep..

[123] Wu T., Liu Y., Yang K., Zhu L., White J.C., Lin D. (2021). Synergistic remediation of PCB-contaminated soil with nanoparticulate zero-valent iron and alfalfa: Targeted changes in the root metabolite-dependent microbial community. Environ. Sci. Nano.

[124] Sakshi, Singh S.K., Haritash A.K. (2019). Polycyclic aromatic hydrocarbons: Soil pollution and remediation. Int. J. Environ. Sci. Technol..

[125] Elgallal M., Fletcher L., Evans B. (2016). Assessment of potential risks associated with chemicals in wastewater used for irrigation in arid and semiarid zones: A review. Agric. Water Manag..

[126] Zhang X., Zhong T., Liu L., Ouyang X. (2015). Impact of soil heavy metal pollution on food safety in China. PLoS ONE.

[127] Karami-Mohajeri S., Abdollahi M. (2011). Toxic influence of organophosphate, carbamate, and organochlorine pesticides on cellular metabolism of lipids, proteins, and carbohydrates: A systematic review. Hum. Exp. Toxicol..

[128] Zhang X., Zhong T., Chen D., Cheng M., Liu L., Zhang X., Li X. (2016). Assessment of arsenic (As) occurrence in arable soil and its related health risk in China. Environ. Geochem. Health.

[129] Shen T., Liu L., Li Y., Wang Q., Dai J., Wang R. (2019). Long-term effects of untreated wastewater on soil bacterial communities. Sci. Total Environ..

[130] Zhang M., Teng Y., Xu Z., Wang J., Christie P., Luo Y. (2016). Cumulative effects of repeated chlorothalonil application on soil microbial activity and community in contrasting soils. J. Soils Sediments.

[131] Zhang C., Zhou T., Zhu L., Juhasz A., Du Z., Li B., Wang J., Wang J., Sun Y. (2019). Response of soil microbes after direct contact with pyraclostrobin in fluvo-aquic soil. Environ. Pollut..

[132] Ren G., Ren W., Teng Y., Li Z. (2015). Evident bacterial community changes but only slight degradation when polluted with pyrene in a red soil. Front. Microbiol..

[133] Estrada G.A., Baldani V.L.D., De Oliveira D.M., Urquiaga S., Baldani J.I. (2013). Selection of phosphate-solubilizing diazotrophic *Herbaspirillum* and *Burkholderia* strains and their effect on rice crop yield and nutrient uptake. Plant Soil.

[134] Defez R., Andreozzi A., Bianco C. (2017). The overproduction of indole-3-acetic acid (IAA) in endophytes upregulates nitrogen fixation in both bacterial cultures and inoculated rice plants. Microb. Ecol..

[135] Xie J.-B., Du Z., Bai L., Tian C., Zhang Y., Xie J.-Y., Wang T., Liu X., Chen X., Cheng Q. (2014). Comparative genomic analysis of N_2_-fixing and non-N_2_-fixing *Paenibacillus* spp.: Organization, evolution and expression of the nitrogen fixation genes. PLoS Genet..

[136] Sarikhani M.R., Oustan S., Ebrahimi M., Aliasgharzad N. (2018). Isolati Isolation and identification of potassium-releasing bacteria in soil and assessment of their ability to release potassium for plants. Eur. J. Soil Sci..

[137] Sun F., Ou Q., Wang N., Guo Z.X., Ou Y., Li N., Peng C. (2020). Isolation and identification of potassium-solubilizing bacteria from *Mikania micrantha* rhizospheric soil and their effect on *M. micrantha* plants. Glob. Ecol. Conserv..

[138] Yaghoubi Khanghahi M., Pirdashti H., Rahimian H., Nematzadeh G., Sepanlou M.G. (2017). Potassium solubilising bacteria (KSB) isolated from rice paddy soil: From isolation, identification to K use efficiency. Symbiosis.

[139] Jiang H., Li S., Wang T., Chi X., Qi P., Chen G. (2021). Interaction between halotolerant phosphate-solubilizing bacteria (*Providencia rettgeri* Strain TPM23) and rock phosphate improves soil biochemical properties and peanut growth in saline soil. Front. Microbiol..

[140] Ji C., Liu Z., Hao L., Song X., Wang C., Liu Y., Li H., Li C., Gao Q., Liu X. (2020). Effects of *Enterobacter cloacae* HG-1 on the nitrogen-fixing community structure of wheat rhizosphere soil and on salt tolerance. Front. Plant Sci..

[141] Allito B.B., Ewusi-Mensah N., Logah V. (2020). Legume-rhizobium strain specificity enhances nutrition and nitrogen fixation in faba bean (*Vicia faba* L.). Agronomy.

[142] Martins M.R., Jantalia C.P., Reis V.M., Döwich I., Polidoro J.C., Alves B.J.R., Boddey R.M., Urquiaga S. (2018). Impact of plant growth-promoting bacteria on grain yield, protein content, and urea-15 N recovery by maize in a Cerrado Oxisol. Plant Soil.

[143] Lu P., Liu H., Liu A. (2019). Biodegradation of dicofol by *Microbacterium* sp. D-2 isolated from pesticide-contaminated agricultural soil. Appl. Biol. Chem..

[144] Hongsawat P., Vangnai A.S. (2011). Biodegradation pathways of chloroanilines by *Acinetobacter baylyi* strain GFJ2. J. Hazard. Mater..

[145] Kamanavalli C.M., Ninnekar H.Z. (2004). Biodegradation of DDT by a *Pseudomonas* species. Curr. Microbiol..

[146] Chanika E., Georgiadou D., Soueref E., Karas P., Karanasios E., Tsiropoulos N.G., Tzortzakakis E.A., Karpouzas D.G. (2011). Isolation of soil bacteria able to hydrolyze both organophosphate and carbamate pesticides. Bioresour. Technol..

[147] Zhang W., Li J., Zhang Y., Wu X., Zhou Z., Huang Y., Zhao Y., Mishra S., Bhatt P., Chen S. (2022). Characterization of a novel glyphosate-degrading bacterial species, *Chryseobacterium* sp. Y16C, and evaluation of its effects on microbial communities in glyphosate-contaminated soil. J. Hazard. Mater..

[148] Gao J., Song P., Wang G., Wang J., Zhu L., Wang J. (2018). Responses of atrazine degradation and native bacterial community in soil to *Arthrobacter* sp. strain HB-5. Ecotoxicol. Environ. Saf..

[149] Sun J., Karuppiah V., Li Y., Pandian S., Kumaran S., Chen J. (2022). Role of cytochrome P450 genes of *Trichoderma atroviride* T23 on the resistance and degradation of dichlorvos. Chemosphere.

[150] Yadav U., Kushwaha S., Anand V., Kumar S., Prakash O., Singh P.C. (2021). Chlorpyrifos degradation by plant growth-promoting *Alcaligenes faecalis* bacteria isolated from oil-contaminated soil. Bioremediat. J..

[151] Ali M., Naqvi T.A., Kanwal M., Rasheed F., Hameed A., Ahmed S. (2012). Detection of the organophosphate degrading gene opdA in the newly isolated bacterial strain *Bacillus pumilus* W1. Ann. Microbiol..

[152] Aswathi A., Pandey A., Sukumaran R.K. (2019). Rapid degradation of the organophosphate pesticide-Chlorpyrifos by a novel strain of *Pseudomonas nitroreducens* AR-3. Bioresour. Technol..

[153] Loh K., Wu T. (2006). Cometabolic transformation of 2-Chlorophenol and 4-Chlorophenol in the presence of phenol by *Pseudomonas putida*. Can. J. Chem. Eng..

[154] Dash D.M., Osborne J.W. (2020). Biodegradation of monocrotophos by a plant growth promoting *Bacillus aryabhattai* (VITNNDJ5) strain in artificially contaminated soil. Int. J. Environ. Sci. Technol..

[155] Li R., Dörfler U., Munch J.C., Schroll R. (2017). Enhanced degradation of isoproturon in an agricultural soil by a *Sphingomonas* sp. strain and a microbial consortium. Chemosphere.

[156] Muendo B.M., Shikuku V.O., Lalah J.O., Getenga Z.M., Wandiga S.O., Rothballer M. (2021). Enhanced degradation of diuron by two *Bacillus* species isolated from diuron contaminated sugarcane and pineapple-cultivated soils in Kenya. Appl. Soil Ecol..

[157] Zhao H., Geng Y., Chen L., Tao K., Hou T. (2013). Biodegradation of cypermethrin by a novel *Catellibacterium* sp. strain CC-5 isolated from contaminated soil. Can. J. Microbiol..

[158] Ahmad K.S. (2020). Environmental contaminant 2-chloro-N-(2,6-diethylphenyl)-N-(methoxymethyl)acetamide remediation via *Xanthomonas axonopodis* and *Aspergillus niger*. Environ. Res..

[159] Zhang H., Zhang Y., Hou Z., Wang X., Wang J., Lu Z., Zhao X., Sun F., Pan H. (2016). Biodegradation potential of deltamethrin by the *Bacillus cereus* strain Y1 in both culture and contaminated soil. Int. Biodeterior. Biodegrad..

[160] Altowayti W., Algaifi H.A., Bakar S.A., Shahir S. (2019). The adsorptive removal of As (III) using biomass of arsenic resistant *Bacillus thuringiensis* strain WS3: Characteristics and modelling studies. Ecotoxicol. Environ. Saf..

[161] Afegbua S.L., Batty L.C. (2019). Effect of plant growth promoting bacterium; *Pseudomonas putida* UW4 inoculation on phytoremediation efficacy of monoculture and mixed culture of selected plant species for PAH and lead spiked soils. Int. J. Phytoremediat..

[162] Hussain A., Amna, Kamran M.A., Javed M.T., Hayat K., Farooq M.A., Ali N., Ali M., Manghwar H., Jan F. (2019). Individual and combinatorial application of *Kocuria rhizophila* and citric acid on phytoextraction of multi-metal contaminated soils by *Glycine max* L.. Environ. Exp. Bot..

[163] Huang H., Zhao Y., Fan L., Jin Q., Yang G., Xu Z. (2020). Improvement of manganese phytoremediation by *Broussonetia papyrifera* with two plant growth promoting (PGP) *Bacillus* species. Chemosphere.

[164] Kumar A., Voropaeva O., Maleva M., Panikovskaya K., Borisova G., Rajkumar M., Bruno L.B. (2021). Bioaugmentation with copper tolerant endophyte *Pseudomonas lurida* strain EOO26 for improved plant growth and copper phytoremediation by *Helianthus annuus*. Chemosphere.

[165] Wani P.A., Wahid S., Singh R., Kehinde A.M. (2018). Antioxidant and chromium reductase assisted chromium (VI) reduction and Cr (III) immobilization by the rhizospheric *Bacillus* helps in the remediation of Cr (VI) and growth promotion of soybean crop. Rhizosphere.

[166] Haider F.U., Coulter J.A., Cheema S.A., Farooq M., Wu J., Zhang R., Shuaijie G., Liqun C. (2021). Co-application of biochar and microorganisms improves soybean performance and remediate cadmium-contaminated soil. Ecotoxicol. Environ. Saf..

[167] Qadir M., Hussain A., Shah M., Lee I.J., Iqbal A., Irshad M., Ismail, Sayyed A., Husna, Ahmad A. (2022). Comparative assessment of chromate bioremediation potential of *Pantoea conspicua* and *Aspergillus niger*. J. Hazard. Mater..

[168] He S., Guo H., He Z., Yang C., Yu T., Chai Q., Lu L. (2018). Interaction of *Lolium perenne* and *Hyphomicrobium* sp. GHH enhances the removal of 17α-ethinyestradiol (EE2) from soil. J. Soils Sediments.

[169] Ping L., Zhang C., Zhang C., Zhu Y., He H., Wu M., Tang T., Li Z., Zhao H. (2014). Isolation and characterization of pyrene and benzo[a]pyrene-degrading *Klebsiella pneumonia* PL1 and its potential use in bioremediation. Appl. Microbiol. Biotechnol..

[170] Smułek W., Sydow M., Zabielska-Matejuk J., Kaczorek E. (2020). Bacteria involved in biodegradation of creosote PAH-A case study of long-term contaminated industrial area. Ecotoxicol. Environ. Saf..

[171] Masakorala K., Yao J., Cai M., Chandankere R., Yuan H., Chen H. (2013). Isolation and characterization of a novel phenanthrene (PHE) degrading strain *Psuedomonas* sp. USTB-RU from petroleum contaminated soil. J. Hazard. Mater..

[172] Cunliffe M., Kertesz M.A. (2006). Effect of *Sphingobium yanoikuyae* B1 inoculation on bacterial community dynamics and polycyclic aromatic hydrocarbon degradation in aged and freshly PAH-contaminated soils. Environ. Pollut..

[173] Wang T., Su D., Wang X., He Z. (2020). Adsorption-degradation of polycyclic aromatic hydrocarbons in soil by immobilized mixed bacteria and its effect on microbial communities. J. Agric. Food Chem..

[174] DeBruyn J.M., Mead T.J., Sayler G.S. (2012). Horizontal transfer of PAH catabolism genes in *Mycobacterium*: Evidence from comparative genomics and isolated pyrene-degrading bacteria. Environ. Sci. Technol..

[175] Dionisi H.M., Chewning C.S., Morgan K.H., Menn F.M., Easter J.P., Sayler G.S. (2004). Abundance of dioxygenase genes similar to *Ralstonia* sp. strain U2 nagAc is correlated with naphthalene concentrations in coal tar-contaminated freshwater sediments. Appl. Environ. Microbiol..

[176] Mishra S., Singh S.N. (2013). Biodegradation of benzo(a)pyrene mediated by catabolic enzymes of bacteria. Int. J. Environ. Sci. Technol..

[177] Zhou Y.Y., Chen D.Z., Zhu R.Y., Chen J.M. (2011). Substrate interactions during the biodegradation of BTEX and THF mixtures by *Pseudomonas oleovorans* DT4. Bioresour. Technol..

[178] Wu X., Gu Y., Wu X., Zhou X., Zhou H., Amanze C., Shen L., Zeng W. (2020). Construction of a tetracycline degrading bacterial consortium and its application evaluation in laboratory-scale soil remediation. Microorganisms.

[179] Gu B., Ju X., Chang J., Ge Y., Vitousek P.M. (2015). Integrated reactive nitrogen budgets and future trends in China. Proc. Natl. Acad. Sci. USA.

[180] Zhang M., He Y., Zhou W., Ai L., Liu H., Chen L., Xie Y. (2021). Effects of continuous cropping of *Codonopsis tangshen* on rhizospheric soil bacterial community as determined by pyrosequencing. Diversity.

[181] Ling D., Huang Q., Ouyang Y. (2010). Impacts of simulated acid rain on soil enzyme activities in a latosol. Ecotoxicol. Environ. Saf..

[182] Coban O., De Deyn G.B., van der Ploeg M. (2022). Soil microbiota as game-changers in restoration of degraded lands. Science.

[183] Majeed A., Muhammad Z., Ahmad H. (2018). Plant growth promoting bacteria: Role in soil improvement, abiotic and biotic stress management of crops. Plant Cell Rep..

[184] Liang C., Amelung W., Lehmann J., Kastner M. (2019). Quantitative assessment of microbial necromass contribution to soil organic matter. Glob. Chang. Biol..

[185] Shahrajabian M.H., Sun W., Cheng Q. (2021). The importance of *Rhizobium*, *Agrobacterium*, *Bradyrhizobium*, *Herbaspirillum*, *Sinorhizobium* in sustainable agricultural production. Not. Bot. Horti Agrobot..

[186] Taha K., Berraho E.B., El Attar I., Dekkiche S., Aurag J., Béna G. (2018). *Rhizobium laguerreae* is the main nitrogen-fixing symbiont of cultivated lentil (*Lens culinaris*) in Morocco. Syst. Appl. Microbiol..

[187] Kaiser J.T., Hu Y., Wiig J.A., Rees D.C., Ribbe M.W. (2011). Structure of precursor-bound NifEN: A nitrogenase FeMo cofactor maturase/insertase. Science.

[188] Nonaka A., Yamamoto H., Kamiya N., Kotani H., Yamakawa H., Tsujimoto R., Fujita Y. (2019). Accessory proteins of the nitrogenase assembly, NifW, NifX/NafY, and NifZ, are essential for diazotrophic growth in the nonheterocystous cyanobacterium *Leptolyngbya boryana*. Front. Microbiol..

[189] Fay A.W., Blank M.A., Rebelein J.G., Lee C.C., Ribbe M.W., Hedman B., Hodgson K.O., Hu Y. (2016). Assembly scaffold NifEN: A structural and functional homolog of the nitrogenase catalytic component. Proc. Natl. Acad. Sci. USA.

[190] Alemneh A.A., Zhou Y., Ryder M.H., Denton M.D. (2020). Mechanisms in plant growth-promoting rhizobacteria that enhance legume-rhizobial symbioses. J. Appl. Microbiol.

[191] Lodwig E.M., Hosie A.H., Bourdes A., Findlay K., Allaway D., Karunakaran R., Downie J.A., Poole P.S. (2003). Amino-acid cycling drives nitrogen fixation in the legume-*Rhizobium* symbiosis. Nature.

[192] Lindstrom K., Mousavi S.A. (2020). Effectiveness of nitrogen fixation in rhizobia. Microb. Biotechnol..

[193] Kim M.-J., Radhakrishnan R., Kang S.-M., You Y.-H., Jeong E.-J., Kim J.-G., Lee I.-J. (2017). Plant growth promoting effect of *Bacillus amyloliquefaciens* H-2-5 on crop plants and influence on physiological changes in soybean under soil salinity. Physiol. Mol. Biol. Plants.

[194] Wu X., Rensing C., Han D., Xiao K.Q., Dai Y., Tang Z., Liesack W., Peng J., Cui Z., Zhang F. (2022). Genome-resolved metagenomics reveals distinct phosphorus acquisition strategies between soil microbiomes. mSystems.

[195] Wu X., Cui Z., Peng J., Zhang F., Liesack W. (2022). Genome-resolved metagenomics identifies the particular genetic traits of phosphate-solubilizing bacteria in agricultural soil. ISME Commun..

[196] Tewari S., Pooniya V., Sharma S. (2020). Next generation bioformulation prepared by amalgamating *Bradyrhizobium*, cell free culture supernatant, and exopolysaccharides enhances the indigenous rhizospheric rhizobial population, nodulation, and productivity of pigeon pea. Appl. Soil Ecol..

[197] Adnan M., Shah Z., Fahad S., Arif M., Alam M., Khan I.A., Mian I.A., Basir A., Ullah H., Arshad M. (2017). Phosphate-solubilizing bacteria nullify the antagonistic effect of soil calcification on bioavailability of phosphorus in alkaline soils. Sci. Rep..

[198] Guo S., Feng B., Xiao C., Wang Q., Chi R. (2021). Phosphate-solubilizing microorganisms to enhance phytoremediation of excess phosphorus pollution in phosphate mining wasteland soil. Bioremediat. J..

[199] Saeed Q., Wang X., Haider F.U., Kucerik J., Mumtaz M.Z., Holatko J., Naseem M., Kintl A., Ejaz M., Naveed M. (2021). Rhizosphere bacteria in plant growth promotion, biocontrol, and bioremediation of contaminated sites: A comprehensive review of effects and mechanisms. Int. J. Mol. Sci..

[200] Zheng W., Morris E.K., Lehmann A., Rillig M.C. (2016). Interplay of soil water repellency, soil aggregation and organic carbon. A meta-analysis. Geoderma.

[201] Rashid M.I., Mujawar L.H., Shahzad T., Almeelbi T., Ismail I.M., Oves M. (2016). Bacteria and fungi can contribute to nutrients bioavailability and aggregate formation in degraded soils. Microbiol. Res..

[202] Caesar-TonThat T.C., Stevens W.B., Sainju U.M., Caesar A.J., West M., Gaskin J.F. (2014). Soil-aggregating bacterial community as affected by irrigation, tillage, and cropping system in the Northern Great Plains. Soil Sci..

[203] Wu Q., He X., Zou Y., He K., Sun Y., Cao M. (2012). Spatial distribution of glomalin-related soil protein and its relationships with root mycorrhization, soil aggregates, carbohydrates, activity of protease and β-glucosidase in the rhizosphere of *Citrus unshiu*. Soil Biol. Biochem..

[204] Henao L.J., Mazeau K. (2009). Molecular modelling studies of clay-exopolysaccharide complexes: Soil aggregation and water retention phenomena. Mater. Sci. Eng. C.

[205] Kallenbach C.M., Frey S.D., Grandy A.S. (2016). Direct evidence for microbial-derived soil organic matter formation and its ecophysiological controls. Nat. Commun..

[206] Six J., Bossuyt H., Degryze S., Denef K. (2004). A history of research on the link between (micro)aggregates, soil biota, and soil organic matter dynamics. Soil Tillage Res..

[207] Yang Y., Xie H., Mao Z., Bao X., He H., Zhang X., Liang C. (2022). Fungi determine increased soil organic carbon more than bacteria through their necromass inputs in conservation tillage croplands. Soil Biol. Biochem..

[208] Xie M., Zhang S., Cui Z., Cao X. (2021). Distribution characteristics and risk assessment of polycyclic aromatic hydrocarbons in soils of a steel enterprise in East China. Bull. Environ. Contam. Toxicol..

[209] Dar M.A., Kaushik G., Villarreal-Chiu J.F. (2019). Pollution status and bioremediation of chlorpyrifos in environmental matrices by the application of bacterial communities: A review. J. Environ. Manag..

[210] Bhadbhade B.J., Sarnaik S.S., Kanekar P.P. (2002). Biomineralization of an organophosphorus pesticide, Monocrotophos, by soil bacteria. J. Appl. Microbiol..

[211] Abraham J., Silambarasan S., Logeswari P. (2014). Simultaneous degradation of organophosphorus and organochlorine pesticides by bacterial consortium. J. Taiwan Inst. Chem. Eng..

[212] Lovecka P., Pacovska I., Stursa P., Vrchotova B., Kochankova L., Demnerova K. (2015). Organochlorinated pesticide degrading microorganisms isolated from contaminated soil. New Biotechnol..

[213] Renault H., Bassard J.E., Hamberger B., Werck-Reichhart D. (2014). Cytochrome P450-mediated metabolic engineering: Current progress and future challenges. Curr. Opin. Plant Biol..

[214] Zhu L., Huo X., Zhou J., Zhang Q., Wang W. (2021). Metabolic activation mechanism of 2,2′,3,3′,6,6′-hexachlorobiphenyl (PCB136) by cytochrome P450 2B6: A QM/MM approach. Sci. Total Environ..

[215] Chadha S., Mehetre S.T., Bansal R., Kuo A., Aerts A., Grigoriev I.V., Druzhinina I.S., Mukherjee P.K. (2018). Genome-wide analysis of cytochrome P450s of *Trichoderma* spp.: Annotation and evolutionary relationships. Fungal Biol. Biotechnol..

[216] Zhang W., Lin Z., Pang S., Bhatt P., Chen S. (2020). Insights into the biodegradation of lindane (gamma-Hexachlorocyclohexane) using a microbial system. Front. Microbiol..

[217] Debruyn J.M., Chewning C.S., Sayler G.S. (2007). Comparative quantitative prevalence of *Mycobacteria* and functionally abundant nidA, nahAc, and nagAc dioxygenase genes in coal tar contaminated sediments. Environ. Sci. Technol..

[218] Nzila A. (2013). Update on the cometabolism of organic pollutants by bacteria. Environ. Pollut..

[219] Aktas O., Cecen F. (2009). Cometabolic bioregeneration of activated carbons loaded with 2-chlorophenol. Bioresour. Technol..

[220] Gu D., Xiang X., Wu Y., Zeng J., Lin X. (2022). Synergy between fungi and bacteria promotes polycyclic aromatic hydrocarbon cometabolism in lignin-amended soil. J. Hazard. Mater..

[221] Zulfiqar U., Farooq M., Hussain S., Maqsood M., Hussain M., Ishfaq M., Ahmad M., Anjum M.Z. (2019). Lead toxicity in plants: Impacts and remediation. J. Environ. Manag..

[222] Redha A., Al-Hasan R., Afzal M. (2021). Synergistic and concentration-dependent toxicity of multiple heavy metals compared with single heavy metals in *Conocarpus lancifolius*. Environ. Sci. Pollut. Res. Int..

[223] Huang X.H., Zhu F., Yan W.D., Chen X.Y., Wang G.J., Wang R.J. (2019). Effects of Pb and Zn toxicity on chlorophyll fluorescence and biomass production of *Koelreuteria paniculata* and *Zelkova schneideriana* young plants. Photosynthetica.

[224] Islam M.S., Kormoker T., Idris A.M., Proshad R., Kabir M.H., Ustaoğlu F. (2021). Plant-microbe-metal interactions for heavy metal bioremediation: A review. Crop Pasture Sci..

[225] Khan I., Ali M., Aftab M., Shakir S., Qayyum S., Haleem K.S., Tauseef I. (2019). Mycoremediation: A treatment for heavy metal-polluted soil using indigenous metallotolerant fungi. Environ. Monit. Assess..

[226] Fakhar A., Gul B., Gurmani A.R., Khan S.M., Ali S., Sultan T., Chaudhary H.J. (2022). Heavy metal remediation and resistance mechanism of *Aeromonas*, *Bacillus*, and *Pseudomonas*: A review. Crit. Rev. Environ. Sci. Technol..

[227] Brady D., Duncan J.R. (1994). Cation loss during accumulation of heavy metal cations by *Saccharomyces cerevisiae*. Biotechnol. Lett..

[228] Khalid M., Ur-Rahman S., Hassani D., Hayat K., Pei Z., Nan H. (2021). Advances in fungal-assisted phytoremediation of heavy metals; a review. Pedosphere.

[229] Dong L., Xu J., Zhang L., Cheng R., Wei G., Su H., Yang J., Qian J., Xu R., Chen S. (2018). Rhizospheric microbial communities are driven by *Panax ginseng* at different growth stages and biocontrol bacteria alleviates replanting mortality. Acta Pharm. Sin. B.

[230] Sun Z., Yang L., Han M., Han Z., Yang L., Cheng L., Yang X., Lv Z.-L. (2019). Biological control ginseng grey mold and plant colonization by antagonistic bacteria isolated from rhizospheric soil of *Panax ginseng* Meyer. Biol. Control.

[231] Lee B.D., Dutta S., Ryu H., Yoo S.J., Suh D.S., Park K. (2015). Induction of systemic resistance in *Panax ginseng* against *Phytophthora cactorum* by native *Bacillus amyloliquefaciens* HK34. J. Ginseng Res..

[232] Durairaj K., Velmurugan P., Park J., Chang W., Park Y., Senthilkumar P., Choi K.-M., Lee J.-H., Oh B.-T. (2018). An investigation of biocontrol activity *Pseudomonas* and *Bacillus* strains against *Panax ginseng* root rot fungal phytopathogens. Biol. Control.

[233] Kang J.P., Huo Y., Yang D.U., Yang D.C. (2021). Influence of the plant growth promoting *Rhizobium panacihumi* on aluminum resistance in *Panax ginseng*. J. Ginseng Res..

[234] Dong L., Xu J., Li Y., Fang H., Niu W., Li X., Zhang Y., Ding W., Chen S. (2018). Manipulation of microbial community in the rhizosphere alleviates the replanting issues in *Panax ginseng*. Soil Biol. Biochem..

[235] Liu N., Shao C., Sun H., Liu Z., Guan Y., Wu L., Zhang L., Pan X., Zhang Z., Zhang Y. (2020). Arbuscular mycorrhizal fungi biofertilizer improves American ginseng (*Panax quinquefolius* L.) growth under the continuous cropping regime. Geoderma.

[236] Amanifar S., Khodabandeloo M., Mohsenifard E., Askari M.S., Ashrafi M. (2019). Alleviation of salt stress and changes in glycyrrhizin accumulation by arbuscular mycorrhiza in liquorice (*Glycyrrhiza glabra*) grown under salinity stress. Environ. Exp. Bot..

[237] Liu J., Wu L., Wei S., Xiao X., Su C., Jiang P., Song Z., Wang T., Yu Z. (2007). Effects of arbuscular mycorrhizal fungi on the growth, nutrient uptake and glycyrrhizin production of licorice (*Glycyrrhiza uralensis* Fisch). Plant Growth Regul..

[238] Yu M., Xie W., Zhang X., Zhang S., Wang Y., Hao Z., Chen B. (2019). Arbuscular mycorrhizal fungi can compensate for the loss of indigenous microbial communities to support the growth of liquorice (*Glycyrrhiza uralensis* Fisch.). Plants.

[239] He C., Wang W., Hou J. (2019). Plant growth and soil microbial impacts of enhancing licorice with inoculating dark septate endophytes under drought stress. Front. Microbiol..

[240] Zhao J., Zhou L., Wu J. (2010). Promotion of *Salvia miltiorrhiza* hairy root growth and tanshinone production by polysaccharide-protein fractions of plant growth-promoting rhizobacterium *Bacillus cereus*. Process Biochem..

[241] Wei X., Cao P., Wang G., Han J. (2020). Microbial inoculant and garbage enzyme reduced cadmium (Cd) uptake in *Salvia miltiorrhiza* (Bge.) under Cd stress. Ecotoxicol. Environ. Saf..

[242] Geneva M.P., Stancheva I.V., Boychinova M.M., Mincheva N.H., Yonova P.A. (2010). Effects of foliar fertilization and arbuscular mycorrhizal colonization on *Salvia officinalis* L. growth, antioxidant capacity, and essential oil composition. J. Sci. Food Agric..

[243] Wang J.W., Zhang Z., Tan R.X. (2001). Stimulation of artemisinin production in *Artemisia annua* hairy roots by the elicitor from the endophytic *Colletotrichum* sp.. Biotechnol. Lett..

[244] Domokos E., Bíró-Janka B., Bálint J., Molnár K., Fazakas C., Jakab-Farkas L., Domokos J., Albert C., Mara G., Balog A. (2020). Arbuscular mycorrhizal fungus rhizophagus irregularis influences *Artemisia annua* plant parameters and artemisinin content under different soil types and cultivation methods. Microorganisms.

[245] Awasthi A., Bharti N., Nair P., Singh R., Shukla A.K., Gupta M.M., Darokar M.P., Kalra A. (2011). Synergistic effect of *Glomus mosseae* and nitrogen fixing *Bacillus subtilis* strain Daz26 on artemisinin content in *Artemisia annua* L.. Appl. Soil Ecol..

[246] Chen J., Li N., Chang J., Ren K., Zhou J., Yang G. (2021). Taxonomic structure of rhizosphere bacterial communities and its association with the accumulation of alkaloidal metabolites in *Sophora flavescens*. Front. Microbiol..

[247] Wu Z., Meng X., Jiao Y., Guo B., Sui X., Ma S., Chen W., Singh R. (2021). *Bradyrhizobium arachidis* mediated enhancement of (oxy)matrine content in the medicinal legume *Sophora flavescens*. Lett. Appl. Microbiol..

[248] Khalilpour M., Mozafari V., Abbaszadeh-Dahaji P. (2021). Tolerance to salinity and drought stresses in pistachio (*Pistacia vera* L.) seedlings inoculated with indigenous stress-tolerant PGPR isolates. Sci. Hortic..

[249] Pankaj U., Singh D.N., Mishra P., Gaur P., Babu C.S.V., Shanker K., Verma R.K. (2020). Autochthonous halotolerant plant growth-promoting rhizobacteria promote bacoside A yield of *Bacopa monnieri* (L.) Nash and phytoextraction of salt-affected soil. Pedosphere.

[250] Attarzadeh M., Balouchi H., Rajaie M., Movahhedi D.M., Salehi A. (2019). Growth and nutrient content of *Echinacea purpurea* as affected by the combination of phosphorus with arbuscular mycorrhizal fungus and *Pseudomonas florescent* bacterium under different irrigation regimes. J. Environ. Manag..

[251] Barnawal D., Maji D., Bharti N., Chanotiya C.S., Kalra A. (2013). ACC deaminase-containing *Bacillus subtilis* reduces stress ethylene-induced damage and improves mycorrhizal colonization and rhizobial nodulation in *Trigonella foenum-graecum* under drought stress. J. Plant Growth Regul..

[252] Dadrasan M., Chaichi M.R., Pourbabaee A.A., Yazdani D., Keshavarz-Afshar R. (2015). Deficit irrigation and biological fertilizer influence on yield and trigonelline production of fenugreek. Ind. Crop. Prod..

[253] Mishra B.K., Meena K.K., Dubey P.N., Aishwath O.P., Kant K., Sorty A.M., Bitla U. (2016). Influence on yield and quality of fennel (*Foeniculum vulgare* Mill.) grown under semi-arid saline soil, due to application of native phosphate solubilizing rhizobacterial isolates. Ecol. Eng..

[254] Tian Y., Liu Y., Yue L., Uwaremwe C., Zhao X., Zhou Q., Wang Y., Wang R. (2022). Bacterial inoculant and sucrose amendments improve the growth of *Rheum palmatum* L. by reprograming its metabolite composition and altering its soil microbial community. Int. J. Mol. Sci..

[255] Benson A., Joe M.M., Karthikeyan B., Sa T., Rajasekaran C. (2014). Role of *Achromobacter xylosoxidans* AUM54 in micropropagation of endangered medicinal plant *Naravelia zeylanica* (L.) DC. J. Plant Growth Regul..

[256] Li Y., Liu Y., Zhang H., Yang Y., Wei G., Li Z. (2021). The composition of root-associated bacteria and fungi of *Astragalus mongholicus* and their relationship with the bioactive ingredients. Front. Microbiol..

[257] Zhang Y., Zheng L., Zheng Y., Xue S., Zhang J., Huang P., Zhao Y., Hao X., He Z., Hu Z. (2020). Insight into the assembly of root-associated microbiome in the medicinal plant *Polygonum cuspidatum*. Ind. Crop. Prod..

[258] Wang Y., Wang M., Li Y., Wu A., Huang J. (2018). Effects of arbuscular mycorrhizal fungi on growth and nitrogen uptake of *Chrysanthemum morifolium* under salt stress. PLoS ONE.

[259] Barnawal D., Pandey S.S., Bharti N., Pandey A., Ray T., Singh S., Chanotiya C.S., Kalra A. (2017). ACC deaminase-containing plant growth-promoting rhizobacteria protect *Papaver somniferum* from downy mildew. J. Appl. Microbiol..

[260] Zhang S., Jiang Q., Liu X., Liu L., Ding W. (2020). Plant growth promoting rhizobacteria alleviate aluminum toxicity and ginger bacterial wilt in acidic continuous cropping soil. Front. Microbiol..

[261] Tripathi P., Yadav R., Das P., Singh A., Singh R.P., Kandasamy P., Kalra A., Khare P. (2021). Endophytic bacterium CIMAP-A7 mediated amelioration of atrazine induced phyto-toxicity in *Andrographis paniculata*. Environ. Pollut..

[262] Khan K., Pankaj U., Verma S.K., Gupta A.K., Singh R.P., Verma R.K. (2015). Bio-inoculants and vermicompost influence on yield, quality of *Andrographis paniculata*, and soil properties. Ind. Crop. Prod..

[263] Lima C.S., Santos H.R.S., Albuquerque U.P.D., Silva F.S.B.D. (2017). Mycorrhizal symbiosis increase the level of total foliar phenols and tannins in *Commiphora leptophloeos* (Mart.) J.B. Gillett seedlings. Ind. Crop. Prod..

[264] Toussaint J., Smith F.A., Smith S.E. (2007). Arbuscular mycorrhizal fungi can induce the production of phytochemicals in sweet basil irrespective of phosphorus nutrition. Mycorrhiza.

[265] Zhou J.Y., Sun K., Chen F., Yuan J., Li X., Dai C.C. (2018). Endophytic *Pseudomonas* induces metabolic flux changes that enhance medicinal sesquiterpenoid accumulation in *Atractylodes lancea*. Plant Physiol. Biochem..

[266] Mañero F.J.G., Algar E., Martín Gómez M.S., Saco Sierra M.D., Solano B.R. (2012). Elicitation of secondary metabolism in *Hypericum perforatum* by rhizosphere bacteria and derived elicitors in seedlings and shoot cultures. Pharm. Biol..

[267] Zubek S., Mielcarek S., Turnau K. (2012). Hypericin and pseudohypericin concentrations of a valuable medicinal plant *Hypericum perforatum* L. are enhanced by arbuscular mycorrhizal fungi. Mycorrhiza.

[268] Ghorbanpour M. (2013). Role of plant growth promoting rhizobacteria on antioxidant enzyme activities and tropane alkaloids production of *Hyoscyamus niger* under water deficit stress. Turk. J. Biol..

[269] Jami N., Rahimi A., Naghizadeh M., Sedaghati E. (2020). Investigating the use of different levels of Mycorrhiza and Vermicompost on quantitative and qualitative yield of saffron (*Crocus sativus* L.). Sci. Hortic..

[270] Bharti N., Barnawal D., Shukla S., Tewari S.K., Katiyar R.S., Kalra A. (2016). Integrated application of *Exiguobacterium oxidotolerans*, *Glomus fasciculatum*, and vermicompost improves growth, yield and quality of *Mentha arvensis* in salt-stressed soils. Ind. Crop. Prod..

[271] Singh S., Tripathi A., Maji D., Awasthi A., Vajpayee P., Kalra A. (2019). Evaluating the potential of combined inoculation of *Trichoderma harzianum* and *Brevibacterium halotolerans* for increased growth and oil yield in *Mentha arvensis* under greenhouse and field conditions. Ind. Crop. Prod..

[272] Liu S., Garcia-Palacios P., Tedersoo L., Guirado E., van der Heijden M., Wagg C., Chen D., Wang Q., Wang J., Singh B.K. (2022). Phylotype diversity within soil fungal functional groups drives ecosystem stability. Nat. Ecol. Evol..

[273] Ling N., Wang T., Kuzyakov Y. (2022). Rhizosphere bacteriome structure and functions. Nat. Commun..

[274] Aslam M.M., Okal E.J., Idris A.L., Qian Z., Xu W., Karanja J.K., Wani S.H., Yuan W. (2022). Rhizosphere microbiomes can regulate plant drought tolerance. Pedosphere.

[275] Wang C., Li Y., Li M., Zhang K., Ma W., Zheng L., Xu H., Cui B., Liu R., Yang Y. (2021). Functional assembly of root-associated microbial consortia improves nutrient efficiency and yield in soybean. J. Integr. Plant Biol..

[276] Oldroyd G.E.D., Leyser O. (2020). A plant’s diet, surviving in a variable nutrient environment. Science.

[277] Li Y., Shao J., Fu Y., Chen Y., Wang H., Xu Z., Feng H., Xun W., Liu Y., Zhang N. (2022). The volatile cedrene from *Trichoderma guizhouense* modulates Arabidopsis root development through auxin transport and signalling. Plant Cell Environ..

[278] Wang S., Chen A., Xie K., Yang X., Luo Z., Chen J., Zeng D., Ren Y., Yang C., Wang L. (2020). Functional analysis of the OsNPF4.5 nitrate transporter reveals a conserved mycorrhizal pathway of nitrogen acquisition in plants. Proc. Natl. Acad. Sci. USA.

[279] Chen C., Wang M., Zhu J., Tang Y., Zhang H., Zhao Q., Jing M., Chen Y., Xu X., Jiang J. (2022). Long-term effect of epigenetic modification in plant-microbe interactions: Modification of DNA methylation induced by plant growth-promoting bacteria mediates promotion process. Microbiome.

[280] Wu H., Qin X., Wang J., Wu L., Chen J., Fan J., Zheng L., Tangtai H., Arafat Y., Lin W. (2019). Rhizosphere responses to environmental conditions in *Radix pseudostellariae* under continuous monoculture regimes. Agric. Ecosyst. Environ..

[281] Wu H., Xia J., Qin X., Wu H., Zhang S., Zhao Y., Rensing C., Lin W. (2020). Underlying mechanism of wild *Radix pseudostellariae* in tolerance to disease under the natural forest cover. Front. Microbiol..

[282] Gauri S.S., Mandal S.M., Dey S., Pati B.R. (2012). Biotransformation of p-coumaric acid and 2,4-dichlorophenoxy acetic acid by *Azotobacter* sp. strain SSB81. Bioresour. Technol..

[283] Zhang B., Weston L.A., Li M., Zhu X., Weston P.A., Feng F., Zhang B., Zhang L., Gu L., Zhang Z. (2020). *Rehmannia glutinosa* replant issues: Root exudate-rhizobiome interactions clearly influence replant success. Front. Microbiol..

[284] Iqbal N., Khan N.A., Ferrante A., Trivellini A., Francini A., Khan M. (2017). Ethylene role in plant growth, development and senescence: Interaction with other phytohormones. Front. Plant Sci..

[285] Sadeghi H., Ghanaatiyan K. (2017). Probing the responses of four chicory ecotypes by ethylene accumulation and growth characteristics under drought stress. Ital. J. Agron..

[286] Brunetti C., Saleem A.R., Della R.G., Emiliani G., De Carlo A., Balestrini R., Khalid A., Mahmood T., Centritto M. (2021). Effects of plant growth-promoting rhizobacteria strains producing ACC deaminase on photosynthesis, isoprene emission, ethylene formation and growth of *Mucuna pruriens* (L.) DC. in response to water deficit. J. Biotechnol..

[287] Glick B.R., Nascimento F.X. (2021). *Pseudomonas* 1-Aminocyclopropane-1-carboxylate (ACC) deaminase and its role in beneficial plant-microbe interactions. Microorganisms.

[288] Ravanbakhsh M., Sasidharan R., Voesenek L.A., Kowalchuk G.A., Jousset A. (2017). ACC deaminase-producing rhizosphere bacteria modulate plant responses to flooding. J. Ecol..

[289] Orhan F. (2021). Potential of halophilic/halotolerant bacteria in enhancing plant growth under salt stress. Curr. Microbiol..

[290] Li Y., Kong D., Fu Y., Sussman M.R., Wu H. (2020). The effect of developmental and environmental factors on secondary metabolites in medicinal plants. Plant Physiol. Biochem..

[291] Liu H., Gu H., Ye C., Guo C., Zhu Y., Huang H., Liu Y., He X., Yang M., Zhu S. (2021). Planting density affects *Panax notoginseng* growth and ginsenoside accumulation by balancing primary and secondary metabolism. Front. Plant Sci..

[292] Abdul M.N., Kumar I.S., Nadarajah K. (2020). Elicitor and receptor molecules: Orchestrators of plant defense and immunity. Int. J. Mol. Sci..

[293] Wang S., Liang W., Lu J., Yao L., Wang J., Gao W. (2020). *Penicillium* sp. YJM-2013 induces ginsenosides biosynthesis in *Panax ginseng* adventitious roots by inducing plant resistance responses. Chin. Herb. Med..

[294] Chamkhi I., Benali T., Aanniz T., El Menyiy N., Guaouguaou F., El Omari N., El-Shazly M., Zengin G., Bouyahya A. (2021). Plant-microbial interaction: The mechanism and the application of microbial elicitor induced secondary metabolites biosynthesis in medicinal plants. Plant Physiol. Biochem..

[295] Ho T.T., Lee J.D., Jeong C.S., Paek K.Y., Park S.Y. (2018). Improvement of biosynthesis and accumulation of bioactive compounds by elicitation in adventitious root cultures of *Polygonum multiflorum*. Appl. Microbiol. Biotechnol..

[296] Zhou X., Wu Y., Wang X., Liu B., Xu H. (2007). Salidroside production by hairy roots of *Rhodiola sachalinensis* obtained after transformation with *Agrobacterium rhizogenes*. Biol. Pharm. Bull..

[297] Zeng Y., Guo L.-P., Chen B., Hao Z., Wang J.-Y., Huang L.-Q., Yang G., Cui X.-M., Yang L., Wu Z.-X. (2013). Arbuscular mycorrhizal symbiosis and active ingredients of medicinal plants: Current research status and prospectives. Mycorrhiza.

[298] Chaudhary T., Shukla P. (2019). Bioinoculants for bioremediation applications and disease resistance: Innovative perspectives. Indian J. Microbiol..

[299] Enebe M.C., Babalola O.O. (2019). The impact of microbes in the orchestration of plants’ resistance to biotic stress: A disease management approach. Appl. Microbiol. Biotechnol..

[300] Gao M., Xiong C., Gao C., Tsui C., Wang M.-M., Zhou X., Zhang A.-M., Cai L. (2021). Disease-induced changes in plant microbiome assembly and functional adaptation. Microbiome.

[301] Pangesti N., Pineda A., Dicke M., van Loon J.J.A., Leiss K., Leiss K. (2015). Variation in plant-mediated interactions between rhizobacteria and caterpillars: Potential role of soil composition. Plant Biol..

[302] Beneduzi A., Ambrosini A., Passaglia L.M. (2012). Plant growth-promoting rhizobacteria (PGPR): Their potential as antagonists and biocontrol agents. Genet. Mol. Biol..

[303] Kloepper J.W., Ryu C., Zhang S. (2004). Induced systemic resistance and promotion of plant growth by *Bacillus* spp.. Phytopathology.

[304] Buttner H., Niehs S.P., Vandelannoote K., Cseresnyes Z., Dose B., Richter I., Gerst R., Figge M.T., Stinear T.P., Pidot S.J. (2021). Bacterial endosymbionts protect beneficial soil fungus from nematode attack. Proc. Natl. Acad. Sci. USA.

[305] Lamdan N.L., Shalaby S., Ziv T., Kenerley C.M., Horwitz B.A. (2015). Secretome of *Trichoderma* interacting with maize roots: Role in induced systemic resistance. Mol. Cell. Proteomics.

[306] Su P., Tan X., Li C., Zhang D., Cheng J., Zhang S., Zhou X., Yan Q., Peng J., Zhang Z. (2017). Photosynthetic bacterium *Rhodopseudomonas palustris* GJ-22 induces systemic resistance against viruses. Microb. Biotechnol..

[307] Patel S., Thaker V., Kuvad R., Saraf M. (2019). Role of lipopolysaccaride extracted from *Alcaligenes faecalis* as elicitor for the induction of plant defense against fusarium wilt. J. Plant Pathol..

[308] Yin C., Casa Vargas J.M., Schlatter D.C., Hagerty C.H., Hulbert S.H., Paulitz T.C. (2021). Rhizosphere community selection reveals bacteria associated with reduced root disease. Microbiome.

[309] Bozsoki Z., Gysel K., Hansen S.B., Lironi D., Kronauer C., Feng F., de Jong N., Vinther M., Kamble M., Thygesen M.B. (2020). Ligand-recognizing motifs in plant LysM receptors are major determinants of specificity. Science.

[310] Yuan X., Hong S., Xiong W., Raza W., Shen Z., Wang B., Li R., Ruan Y., Shen Q., Dini-Andreote F. (2021). Development of fungal-mediated soil suppressiveness against *Fusarium* wilt disease via plant residue manipulation. Microbiome.

[311] Kim D.R., Jeon C.W., Cho G., Thomashow L.S., Weller D.M., Paik M.J., Lee Y.B., Kwak Y.-S. (2021). Glutamic acid reshapes the plant microbiota to protect plants against pathogens. Microbiome.

[312] Zhong Y., Xun W., Wang X., Tian S., Zhang Y., Li D., Zhou Y., Qin Y., Zhang B., Zhao G. (2022). Root-secreted bitter triterpene modulates the rhizosphere microbiota to improve plant fitness. Nat. Plants.

[313] Santoyo G. (2021). How plants recruit their microbiome? New insights into beneficial interactions. J. Adv. Res..

[314] Wu H., Wu H., Jiao Y., Zhang Z., Rensing C., Lin W. (2022). The combination of biochar and PGPBs stimulates the differentiation in rhizosphere soil microbiome and metabolites to suppress soil-borne pathogens under consecutive monoculture regimes. GCB Bioenergy.

[315] Zhang Y., Gao X., Shen Z., Zhu C., Jiao Z., Li R., Shen Q. (2019). Pre-colonization of PGPR triggers rhizosphere microbiota succession associated with crop yield enhancement. Plant Soil.

[316] Fira D., Dimkić I., Berić T., Lozo J., Stanković S. (2018). Biological control of plant pathogens by *Bacillus* species. J. Biotechnol..

[317] Jiang G., Zhang Y., Gan G., Li W., Wan W., Jiang Y., Yang T., Zhang Y., Xu Y., Wang Y. (2022). Exploring rhizo-microbiome transplants as a tool for protective plant-microbiome manipulation. ISME Commun..

[318] Wang M., Li S., Chen S., Meng N., Li X., Zheng H., Zhao C., Wang D. (2019). Manipulation of the rhizosphere bacterial community by biofertilizers is associated with mitigation of cadmium phytotoxicity. Sci. Total. Environ..

[319] Shabaan M., Asghar H.N., Akhtar M.J., Ali Q., Ejaz M. (2021). Role of plant growth promoting rhizobacteria in the alleviation of lead toxicity to *Pisum sativum* L.. Int. J. Phytoremediat..

[320] de Fatima P.D., Barbosa M.V., Dos S.J., Pinto F.A., Siqueira J.O., Carneiro M. (2018). Arbuscular mycorrhizal fungi favor the initial growth of *Acacia mangium*, *Sorghum bicolor*, and *Urochloa brizantha* in soil contaminated with Zn, Cu, Pb, and Cd. Bull. Environ. Contam. Toxicol..

[321] Oliveira K.O., Silva A.R.M., da Silva B.F., Milagre H.M.S., Milagre C.D.F. (2018). Insights into the microbial degradation pathways of the ioxynil octanoate herbicide. Biocatal. Agric. Biotechnol..

[322] Pan X., Wang S., Shi N., Fang H., Yu Y. (2018). Biodegradation and detoxification of chlorimuron-ethyl by *Enterobacter ludwigii* sp. CE-1. Ecotoxicol. Environ. Saf..

[323] Gupta M., Mathur S., Sharma T.K., Rana M., Gairola A., Navani N.K., Pathania R. (2016). A study on metabolic prowess of *Pseudomonas* sp. RPT 52 to degrade imidacloprid, endosulfan and coragen. J. Hazard. Mater..

[324] Li H., Qiu Y., Yao T., Ma Y., Zhang H., Yang X., Li C. (2020). Evaluation of seven chemical pesticides by mixed microbial culture (PCS-1): Degradation ability, microbial community, and *Medicago sativa* phytotoxicity. J. Hazard. Mater..

[325] Abdelhameed R.E., Metwally R.A. (2019). Alleviation of cadmium stress by arbuscular mycorrhizal symbiosis. Int. J. Phytoremediat..

[326] Yue Z., Chen Y., Chen C., Ma K., Tian E., Wang Y., Liu H., Sun Z. (2021). Endophytic *Bacillus altitudinis* WR10 alleviates Cu toxicity in wheat by augmenting reactive oxygen species scavenging and phenylpropanoid biosynthesis. J. Hazard. Mater..

[327] Chen B., Xiao X., Zhu Y.G., Smith F.A., Xie Z.M., Smith S.E. (2007). The arbuscular mycorrhizal fungus *Glomus mosseae* gives contradictory effects on phosphorus and arsenic acquisition by *Medicago sativa* Linn. Sci. Total Environ..

[328] Wang Z., Chen Z., Kowalchuk G.A., Xu Z., Fu X., Kuramae E.E. (2021). Succession of the resident soil microbial community in response to periodic inoculations. Appl. Environ. Microbiol..

[329] Morya R., Salvachua D., Thakur I.S. (2020). *Burkholderia*: An untapped but promising bacterial genus for the conversion of aromatic compounds. Trends Biotechnol..

[330] Hayat K., Menhas S., Bundschuh J., Zhou P., Niazi N.K., Amna, Hussain A., Hayat S., Ali H., Wang J. (2020). Plant growth promotion and enhanced uptake of Cd by combinatorial application of *Bacillus pumilus* and EDTA on *Zea mays* L.. Int. J. Phytoremediat..

[331] Li S., Zhang N., Zhang Z., Luo J., Shen B., Zhang R., Shen Q. (2012). Antagonist *Bacillus subtilis* HJ5 controls Verticillium wilt of cotton by root colonization and biofilm formation. Biol. Fertil. Soils.

[332] Sun X., Xu Z., Xie J., Hesselberg-Thomsen V., Tan T., Zheng D., Strube M.L., Dragoš A., Shen Q., Zhang R. (2022). *Bacillus velezensis* stimulates resident rhizosphere *Pseudomonas stutzeri* for plant health through metabolic interactions. ISME J..

[333] Dan A., Zhang N., Qiu R., Li C., Wang S., Ni Z. (2020). Accelerated biodegradation of *p-tert*-butylphenol in the *Phragmites australis* rhizosphere by phenolic root exudates. Environ. Exp. Bot..

[334] Xia L., Miao Y., Cao A., Liu Y., Liu Z., Sun X., Xue Y., Xu Z., Xun W., Shen Q. (2022). Biosynthetic gene cluster profiling predicts the positive association between antagonism and phylogeny in *Bacillus*. Nat. Commun..

